# Instrumental Activities of Daily Living as a Mediator Between Cognition and Anti-Saccade Uncorrected Error Rate in CSVD: An Exploratory Analysis with Education Moderation Testing

**DOI:** 10.3390/brainsci16101137

**Published:** 2026-10-21

**Authors:** Xuan Xu, Kaige Zhang, Luxuan Xia, Xiaoxu Liu, Liye Wang, Yilan Zheng, Chengru Jia, Nannan Li, Zhigang Chen

**Affiliations:** 1 Department of Neurology I, Dongfang Hospital, Beijing University of Chinese Medicine, Beijing 100078, China; xx1755013900@126.com (X.X.); zhangkaige2000@163.com (K.Z.); wangliye2013@126.com (L.W.); zheng1lan@163.com (Y.Z.); 13258673262@163.com (C.J.); dfyylnn@126.com (N.L.); 2 Department of Neurology I, Dongzhimen Hospital, Beijing University of Chinese Medicine, Beijing 100700, China; xialuxvan@163.com; 3 Department of Ophthalmology, Dongfang Hospital, Beijing University of Chinese Medicine, Beijing 100078, China; 19861403262@163.com

**Keywords:** cerebral small vessel disease, instrumental activities of daily living, anti-saccade, cognitive reserve, years of education

## Abstract

**Highlights:**

**What are the main findings?**

- In the uncorrected analysis, IADL partially mediated the MoCA–anti-saccade uncorrected error rate association in CSVD (95% BootCI [−1.138, −0.003]), but this effect did not survive FDR correction (BH-corrected *p* = 0.212). It should be noted that the unadjusted bootstrap *p* value was already greater than 0.05; therefore, its non-significance was not solely attributable to FDR multiplicity correction. Moreover, this *p* value was based on a normal approximation and is provided for exploratory reference only, with interval inference for the indirect effect relying primarily on the bootstrap confidence interval.
- The indirect effects for the corrected error rate and correct rate were also non-significant after FDR correction; no formal between-outcome comparison was performed, so outcome-specific mediation cannot be inferred.
- Years of education did not moderate the overall indirect effect: all moderated mediation indices were non-significant, and the Model 14 interaction term lost significance after FDR correction (BH-corrected *p* = 0.051).

**What are the implications of the main findings?**

- The link between the anti-saccade uncorrected error rate and prefrontal–basal ganglia inhibitory function in CSVD remains a cross-sectional exploratory observation, pending validation in independent longitudinal samples.
- IADL may be a candidate mediator linking cognitive decline to impaired executive control, but its directionality and potential as an intervention target require longitudinal and interventional testing.
- The path-specific moderating role of education should be treated as an exploratory trend requiring further validation.

**Abstract:**

**Background**: Cerebral small vessel disease (CSVD) is one of the major causes of cognitive impairment and functional decline in older adults. Instrumental activities of daily living (IADL) may serve as a bridge between cognitive impairment and more basic executive control; however, whether IADL mediates the association between global cognition and anti-saccade inhibitory control in patients with CSVD remains unclear. This study examined the mediating role of IADL between the Montreal Cognitive Assessment (MoCA) and anti-saccade metrics, and exploratorily tested whether years of education moderated this pathway. **Methods**: A total of 171 patients with CSVD were enrolled. The anti-saccade uncorrected error rate, corrected error rate, and correct rate were collected. Using IADL as the mediator, MoCA as the independent variable, and relevant factors as covariates, mediation and moderated mediation analyses were conducted with PROCESS Model 4, Model 7, and Model 14. The same model specifications were applied to all three anti-saccade outcomes. Benjamini–Hochberg FDR correction was used to address multiplicity across outcome screening and interaction tests. Because no formal direct comparisons of indirect effects among the three outcomes were performed, differences in statistical significance across outcomes were not interpreted as evidence of outcome-specific mediation or distinct neural mechanisms. **Results**: (1) In Model 4: in the uncorrected analysis, the indirect effect of IADL between MoCA and the anti-saccade uncorrected error rate was significant (95% BootCI [−1.138, −0.003]); however, after Benjamini–Hochberg FDR correction, this indirect effect was no longer significant (Bootstrap *p* = 0.153, BH-corrected *p* = 0.212). It should be noted that the unadjusted bootstrap *p* value was already greater than 0.05; therefore, its non-significance was not solely attributable to FDR multiplicity correction. The indirect effects for the anti-saccade corrected error rate and correct rate remained non-significant after FDR correction (both BH-corrected *p* = 0.212); no direct comparisons among outcomes were performed in this study. (2) In Model 14: the IADL × years of education interaction term for the uncorrected error rate was significant before correction (*p* = 0.034), but was no longer significant after FDR correction (BH-corrected *p* = 0.051). More importantly, the moderated mediation indices for the uncorrected error rate, corrected error rate, and correct rate were all non-significant (uncorrected error rate: 95% BootCI [−0.021, 0.123]; corrected error rate: 95% BootCI [−0.186, 0.016]; correct rate: 95% BootCI [−0.052, 0.119]), suggesting that years of education did not significantly moderate the overall indirect effect. (3) HC3 robust standard error sensitivity analysis showed that in the uncorrected error rate model, path a in Model 4 was no longer significant (*p* = 0.077), and the Bootstrap 95% confidence interval for the indirect effect was [−1.132, −0.002], with its upper limit close to 0; in Model 14, the IADL × years of education interaction term was also no longer significant (*p* = 0.211), indicating limited robustness of the above indirect association. **Conclusions**: In this exploratory cross-sectional analysis, IADL may partially mediate the association between global cognition and the anti-saccade uncorrected error rate in patients with CSVD before correction; however, this indirect effect was not statistically supported after multiplicity correction. It should be emphasized that the unadjusted bootstrap *p* value was itself non-significant, and this *p* value was based on a normal approximation and serves as an exploratory reference only; after considering the robustness of the bootstrap confidence interval, this indirect effect did not reach statistical robustness. The findings of this study did not provide evidence for outcome-specific mediation, and the moderated mediation effect of years of education was not statistically supported. These results should be interpreted as exploratory associations and require further validation in independent longitudinal samples.

## 1. Introduction

Cerebral small vessel disease (CSVD) is one of the most common cerebrovascular diseases in older adults. Its neuroimaging manifestations include white matter hyperintensities (WMH), lacunar infarcts, cerebral microbleeds, and enlarged perivascular spaces [1]. With population aging, the prevalence of CSVD continues to rise, and it has become an important cause of cognitive impairment and decline in activities of daily living in older adults [2]. CSVD accounts for about 25% of all strokes, and its imaging detection rate in people over 90 years old is almost close to 100% [3]. More importantly, CSVD is not merely a general vascular risk factor; it has profound clinical significance—it is closely related to more severe cognitive impairment [4], a higher risk of dementia conversion [5], and worse prognosis [6]. However, previous studies have mostly focused on describing the epidemiological associations between CSVD and cognitive impairment, dementia conversion, and functional decline, while direct testing of the specific mediating pathways linking global cognition to more basic executive control processes is still lacking. Therefore, this study directly tested in patients with CSVD whether daily functioning mediates the association between cognition and anti-saccade indicators, and exploratorily examined whether years of education moderates this mediating pathway.

Cognitive impairment caused by CSVD can manifest in multiple domains, such as executive dysfunction, slowed information processing speed, decreased attention, and memory decline. Among these, executive dysfunction and decreased attention are considered core features of CSVD-related cognitive impairment [7]. Neuroimaging studies have shown that CSVD can disrupt the integrity of cortical-subcortical networks, especially prefrontal connectivity, leading to dysfunction of cognitive networks [8,9]. Recent studies have further elucidated the pathophysiological mechanisms of cognitive impairment in CSVD, emphasizing the key roles of white matter microstructural damage [10], reduced cerebral perfusion [11], and neurovascular unit dysfunction [12]. Although these associations have been confirmed, there is still insufficient research on how cognitive impairment in patients with CSVD affects their daily functioning through psychological and behavioral factors.

Instrumental activities of daily living (IADL) include complex activities such as financial management, medication management, use of transportation, shopping, and housework, and are key indicators reflecting an individual’s ability to live independently in the community [13]. From a neuropsychological perspective, the effective performance of IADL depends on the coordinated operation of multiple cognitive domains, especially executive function [14]. Previous studies have mostly regarded IADL as a functional outcome of cognitive impairment. For example, Nagaoka et al. found that IADL mediates the association between cognitive function and depressive symptoms [15]. In a study of older adults with comorbid diabetes and hypertension, IADL and regular exercise had independent or chain-mediating effects on the relationship between physical performance and cognitive function [16]. Jokinen et al. also showed that the association between WMH volume and IADL was mediated by global cognitive function, and that WMH and IADL were significantly related to multiple subdomains of cognitive assessment [17,18]. However, these studies mainly support the direction of “cognitive impairment → IADL decline.” Global cognitive assessment largely reflects the comprehensive level of cognitive function, whereas anti-saccade inhibitory control is a more basic executive control indicator closer to the level of neural circuits. IADL, as an embodiment of integrated daily functioning, may lie between the two. This hypothesis does not deny that IADL itself depends on executive function; rather, it regards IADL as an exploratory mediating link between global cognition and objective executive control performance.

The anti-saccade task is a classic experimental paradigm for measuring inhibitory oculomotor control in humans. In this task, participants need to inhibit the dominant reflexive saccade toward a suddenly appearing peripheral visual stimulus (pro-saccade) and instead make a voluntary saccade in the opposite direction [19]. Successful anti-saccade performance depends on the integrity of the prefrontal-basal ganglia circuit [20]. Studies suggest that different types of anti-saccade errors may involve different cognitive processes: correctable errors and uncorrected errors show a dissociated pattern in psychological associations. The former is not significantly correlated with spatial memory and the Stroop test, whereas the latter is significantly correlated, suggesting that uncorrected errors may involve a failure to maintain task goals in working memory, while corrected errors may reflect active correction after an initial inhibition failure [21,22,23]. Previous studies have pointed out that patients with WMH show abnormal anti-saccade performance, especially in frontal aspects [24]. Chen et al. found that anti-saccade accuracy, stride speed, and swing speed were significantly correlated with cognitive impairment in CSVD, and this oculo-gait pattern had good efficacy in identifying CSVD cognitive impairment [25]. However, research on the factors affecting different anti-saccade indicators and their pathways with cognition and function is still relatively scarce. Considering that cognitive impairment in CSVD mainly arises from disruption of cortical-subcortical connections [7,26], this study speculated that cognitive impairment in CSVD may be indirectly related to IADL decline and inhibitory control function represented by anti-saccade uncorrected error rate; however, existing evidence is not sufficient to clearly attribute uncorrected error rate to a specific neural pathway (such as the “direct pathway”), and the neural substrate distinction between corrected and uncorrected errors remains to be verified. In addition, the three anti-saccade indicators differ in denominator, direction, and psychometric meaning, and whether their associations with cognitive function and IADL are consistent still needs to be compared in parallel within the same model framework. It should be particularly noted that a significant indirect effect for one outcome and a non-significant one for another cannot directly infer that the indirect effects differ between the two. Therefore, this study did not presuppose that a certain anti-saccade indicator has outcome-specific mediation, but included all three indicators in the analysis and reported their respective results.

Years of education is one of the most commonly used proxies for cognitive reserve [27]. Cognitive reserve reflects the brain’s ability to maintain cognitive function in the face of pathological changes and is influenced by developmental, environmental, and psychosocial factors [28]. Because cognitive reserve may vary with educational background, examining its moderating role in the CSVD population is particularly important [29]. Related evidence shows that educational level is significantly correlated with the degree of cognitive impairment in CSVD [30], and the protective effect of education on cognitive decline clearly exists in early amnestic mild cognitive impairment (aMCI) [31,32]. Jokinen et al. found that higher education and occupational attainment were significantly correlated with baseline cognitive scores and predicted a slower rate of cognitive decline during follow-up, suggesting that education can buffer the harmful effects of WMH on cognition [33]. Li et al. divided patients with CSVD into low cognitive reserve and high cognitive reserve groups according to years of education, collected relevant MRI and neuropsychological assessment results, and found that cognitive reserve may delay cognitive decline in patients with CSVD by protecting white matter fibers (especially the left arcuate fasciculus) [34]. However, research on whether education moderates the behavioral chain of “cognition → IADL → oculomotor inhibition” is still limited. According to cognitive reserve theory [28], the protective effect of education may be manifested after functional impairment, rather than in the early stage of the transition from cognition to function. Therefore, this study exploratorily examined whether education may specifically moderate the latter path of “IADL → anti-saccade uncorrected error rate,” rather than the first path of “MoCA → IADL.” It should be clarified that testing the interaction term of the latter path and testing “moderated mediation” are two different hypotheses: the former addresses whether the conditional association of a specific path varies with years of education, while the latter addresses whether the indirect effect itself varies with years of education. This study tested and reported them separately, and did not infer that moderated mediation is established based on a significant path interaction term. This moderation hypothesis remains an exploratory test within a cross-sectional association framework.

Based on the above theoretical background, this study proposed the following theory-oriented research questions and exploratory hypotheses: (1) Does IADL play a mediating role between MoCA and anti-saccade uncorrected error rate? (2) Is the moderating role of education manifested at the level of conditional association of a specific path, and is moderated mediation statistically supported? (3) Do the three anti-saccade indicators (uncorrected error rate, corrected error rate, correct rate) differ in their associations with cognitive function and IADL? It should be noted that, because previous studies have no consistent conclusion on the relationship between the three anti-saccade indicators and cognitive function, this study included all three indicators in the analysis and compared them using the same model specification; in the subsequent mediation and moderation analyses, anti-saccade uncorrected error rate was used as the main oculomotor executive function indicator. This choice combined theoretical background and preliminary statistical results and was an exploratory analysis, rather than a pre-determined sole primary outcome. Based on this, this study did not interpret “significant indirect effect for uncorrected error rate, non-significant for corrected error rate and correct rate” as evidence of outcome-specific mediation or different neural mechanisms, unless a direct comparison between outcomes was separately conducted. Therefore, the objectives of this study included: ① comparing the relationships of the three anti-saccade indicators with executive function and daily activity ability; ② testing, within an exploratory framework, whether uncorrected error rate mediates or moderates the relevant relationships; ③ reporting the results of the three indicators under a consistent model and correcting for multiple comparisons. In the moderation analysis section, the latter-path interaction term and the moderated mediation index were reported separately, and it was clearly stated whether the latter was statistically supported. Given that the role of education in cognitive impairment remains controversial [35,36], and that the global aging burden brought by CSVD is enormous [2], clarifying this association pattern may help develop targeted intervention measures, especially for patients with CSVD with low educational levels, to enhance their functional reserve; however, the relevant intervention effects still need to be verified in longitudinal or interventional studies.

## 2. Methods

### 2.1. Data Acquisition

#### 2.1.1. Participant Recruitment and Initial Screening

Participants were all from Dongfang Hospital, Beijing University of Chinese Medicine, enrolled between January 2025 and July 2026, using a consecutive enrollment method. Inclusion criteria were as follows: CSVD diagnosis meeting the international neuroimaging standards for CSVD 2023 (STRIVE-2) [37]; age 40–85 years; normal or corrected-to-normal vision; voluntary cooperation with this study and ability to sign informed consent. Exclusion criteria: cognitive decline confirmed to be caused by other reasons such as large vessel disease, cardioembolic cerebral embolism, or strategic infarcts; organic brain disease, mental disorders, or major malignant diseases; illiteracy. This study followed the principles of the Declaration of Helsinki and was approved by the Ethics Committee of Dongfang Hospital, Beijing University of Chinese Medicine (JDF-IRB-2024033002).

In the initial screening stage, basic demographic information such as sex, age, and years of education was collected. Subsequently, HAMA [38] and HAMD [39] were collected only to exclude those with severe anxiety/depression (HAMA ≥ 29 or HAMD ≥ 25). The IADL [40] was used to assess patients’ instrumental activities of daily living. This scale contains 8 items (telephone use, shopping, food preparation, housekeeping, laundry, transportation, medication management, and finances), with a total score range of 0 to 24. Higher scores indicate better instrumental activities of daily living. The assessment was completed by trained neurologists through face-to-face interviews, and the relevant information was verified by informants.

#### 2.1.2. Secondary Screening: Cognitive Testing

Cognitive function was mainly assessed using the Beijing version of the Montreal Cognitive Assessment (MoCA) [41]. This study used an education-corrected definition of cognitive impairment: 1 point was added for participants with years of education ≤ 12 to correct for the influence of education, and an education-corrected MoCA score < 26 was defined as the presence of cognitive impairment.

#### 2.1.3. Eye Movement Data Collection

The EyeKnow intelligent eye-tracking analysis and evaluation system (Beijing CAS-Ruiyi Information Technology Co., Ltd., Beijing, China) [25] was used to collect the anti-saccade oculomotor paradigm. The eye-tracking assessor was blinded to cognitive or clinical information. The EyeKnow intelligent eye-tracking analysis and evaluation system consists of a tablet computer and a virtual reality headset equipped with eye-tracking cameras. This device presents stimuli on an integrated monitor of the eye-tracking system and has an embedded data processing module to analyze and compute eye movement parameters. The system uses an infrared dual-acquisition module, with a sampling rate of 120 Hz, and is equipped with a 3664 × 1920 pixel high-resolution display with a vertical refresh rate of 120 Hz and high visual clarity. During eye-tracking data acquisition, the pupil-corneal tracking technique was used, and deep learning image recognition algorithms were applied to the acquired images. During the process of participants performing eye movement test paradigms such as anti-saccades, eye movement data were collected, and various quantitative data such as eye movement correct rate and corrected error rate were automatically recorded and provided. This enabled dynamic capture and recording of the participants’ eye movements throughout the process and provided quantitative parameters of eye movement-related behavior, describing key indicators of eye movement from temporal and spatial dimensions. Before the formal test, a nine-point calibration technique was used to achieve a maximum calibration radius error ≤ 2°. Subsequently, each guided pre-experimental task was performed to ensure that each participant correctly understood and performed the task.

The target stimulus presentation duration of the anti-saccade task was 1200 ms. This 1200 ms window served both as the detection window for the initial saccade and as the time window for accepting corrective saccades. The accepted range of saccade latency was ≥80 ms; saccades with latency < 80 ms were regarded as anticipatory/invalid saccades, excluded according to the exclusion criteria, and not included in valid trial analysis. If no valid saccade was completed within the 1200 ms target presentation window, the trial was counted as unsuccessful/no-response timeout. Trial classification rules were: (1) correct anti-saccade: the initial saccade was immediately directed opposite to the target and completed within 1200 ms; (2) error saccade followed by effective correction: the initial saccade was directed toward the target (wrong direction), but a corrective saccade was performed within the 1200 ms target presentation window and landed in the correct contralateral region; (3) uncorrected error: the initial saccade was directed toward the target (wrong direction), and no effective correction reaching the correct contralateral trigger region was completed within the 1200 ms target presentation window; (4) no response/timeout: no saccade was made within the 1200 ms target presentation window; saccades with latency < 80 ms were anticipatory saccades and were not counted as valid trials according to the exclusion criteria. Timeout no-response trials and anticipatory saccade trials were not counted as valid anti-saccade trials. If a trial could not be judged for initial saccade or corrective saccade due to loss of eye movement signal, calibration drift, or inability to reliably identify saccade direction, the trial was excluded as a tracking failure/invalid trial and did not enter the numerator or denominator of any indicator.

The definitions of the indicators were as follows: the numerator of the anti-saccade correct rate was the number of correct anti-saccade trials in which the initial saccade was immediately directed opposite to the target and completed within 1200 ms, and the denominator was the number of valid anti-saccade trials; the numerator of the anti-saccade uncorrected error rate was the number of trials in which the initial saccade was directed toward the target (wrong direction) and no effective correction reaching the correct contralateral trigger region was completed within 1200 ms, and the denominator was the number of valid anti-saccade trials; the anti-saccade corrected error rate was conditioned on valid anti-saccade trials with an initial wrong-direction saccade, and its numerator was the number of trials in which the initial saccade direction was wrong but a corrective saccade was completed within 1200 ms and landed in the correct contralateral trigger region, and its denominator was the number of valid anti-saccade trials with an initial wrong-direction saccade. Therefore, the correct rate and uncorrected error rate had the same denominator, while the corrected error rate used a conditional denominator; the three percentages did not share the same denominator, and a sum exceeding 100% was due to different denominators. If a participant had no initial wrong-direction trials among valid anti-saccade trials, their corrected error rate could not be defined and was treated as a missing value, not recorded as 0% or 100%.

In the final screening, this study excluded a total of 10 cases, all due to poor cooperation with eye-tracking acquisition. Among the 171 patients finally included in the analysis, both correct rate and uncorrected error rate could be calculated; 163 had initial wrong-direction trials among valid anti-saccade trials and could have corrected error rate calculated, while the remaining 8 had no initial wrong-direction trials, so corrected error rate was treated as a missing value and not included in individual-level statistics for corrected error rate. Except for the structural missingness of corrected error rate, there was no missing data for other key variables. All three anti-saccade indicators were pre-collected and included in the initial analysis; the selection process of the main oculomotor indicator for subsequent mediation and moderation analyses was an exploratory analysis.

### 2.2. Data Analysis

All statistical analyses were performed using SPSS 27.0 (IBM Corp., Armonk, NY, USA) and the PROCESS 4.2 macro (Andrew F. Hayes). First, descriptive statistics were performed for key variables; continuous variables were expressed as mean ± standard deviation, and categorical variables as frequency (constituent ratio).

Before the formal mediation analysis, this study described the correlations of the three anti-saccade indicators and used the same model specification to conduct mediation effect tests (PROCESS 4.2 Model 4) and moderation effect tests (PROCESS 4.2 Model 7, Model 14) for the three indicators separately. The anti-saccade corrected error rate results are shown inthe main text, and the results for corrected error rate and correct rate are also reported in the Supplementary Materials (Tables S1–S5). Based on theoretical background and preliminary statistical screening results, subsequent mediation and moderated mediation analyses used “anti-saccade uncorrected error rate” as the main oculomotor executive function indicator. It should be emphasized that this outcome selection process was an exploratory analysis, rather than a pre-set confirmatory primary outcome. Finally, Benjamini–Hochberg FDR correction was used to address multiplicity issues arising from outcome screening and interaction testing.

The education-corrected scores of MoCA were used in this study. To evaluate the impact of incorporating education into the predictor definitions, a sensitivity analysis using the original (uncorrected) MoCA scores was conducted, and the results are reported in the Supplementary Materials (Tables S6–S8). To describe sample characteristics, we used Pearson correlation analysis to examine the associations of age and years of education with anti-saccade uncorrected error rate, and independent-samples *t*-tests to compare the effects of sex and vascular risk factors on uncorrected error rate. The results showed that years of education was significantly negatively correlated with uncorrected error rate (*p* < 0.05), while differences in age, sex, and vascular risk factors did not reach significance (*p* > 0.05). Further considering clinical practice, years of education, age, and vascular risk factors were controlled as covariates in subsequent mediation models. The core exploration of this study concerned the cross-sectional associations among MoCA, IADL, and anti-saccade metrics. HAMD/HAMA were used in this study only for initial screening of severe anxiety/depression and constituted part of the sample selection criteria, rather than baseline confounders collected for adjustment purposes. The analytical sample was therefore conditional on this screening process; moreover, HAMD/HAMA were measured concurrently with the core variables, so it cannot be established that they preceded the exposure and outcome. Clinically, they are more likely to partially reflect downstream consequences or shared outcomes of cognitive/IADL/oculomotor abnormalities. Including them as ordinary covariates in the main models could introduce selection bias or overadjustment bias. Therefore, the main models did not include HAMD/HAMA and instead included only the prespecified conventional clinical adjustment variables (age, educational level and vascular risk factors). For transparency and robustness, we have additionally conducted sensitivity analyses in which HAMD/HAMA were further included in the models, provided in the Supplementary Materials (Tables S9–S11). It should be noted that in the Model 4 mediation model, years of education was controlled as a covariate; in the Model 7 and Model 14 moderated mediation models, years of education was included as a moderator in the interaction term and no longer simultaneously as an ordinary covariate.

Given that the anti-saccade uncorrected error rate is a percentage indicator with zero inflation and a right-skewed distribution, this study performed residual diagnostics on the regression models before the formal mediation analysis. If residual diagnostics suggested heteroscedasticity or non-normality, HC3 heteroscedasticity-robust standard errors were used to re-estimate the regression coefficients, and the results under ordinary least squares and robust standard errors were compared. Indirect effects were still obtained using Bootstrap 5000 times to obtain percentile bootstrap confidence intervals.

**Figure 1:**
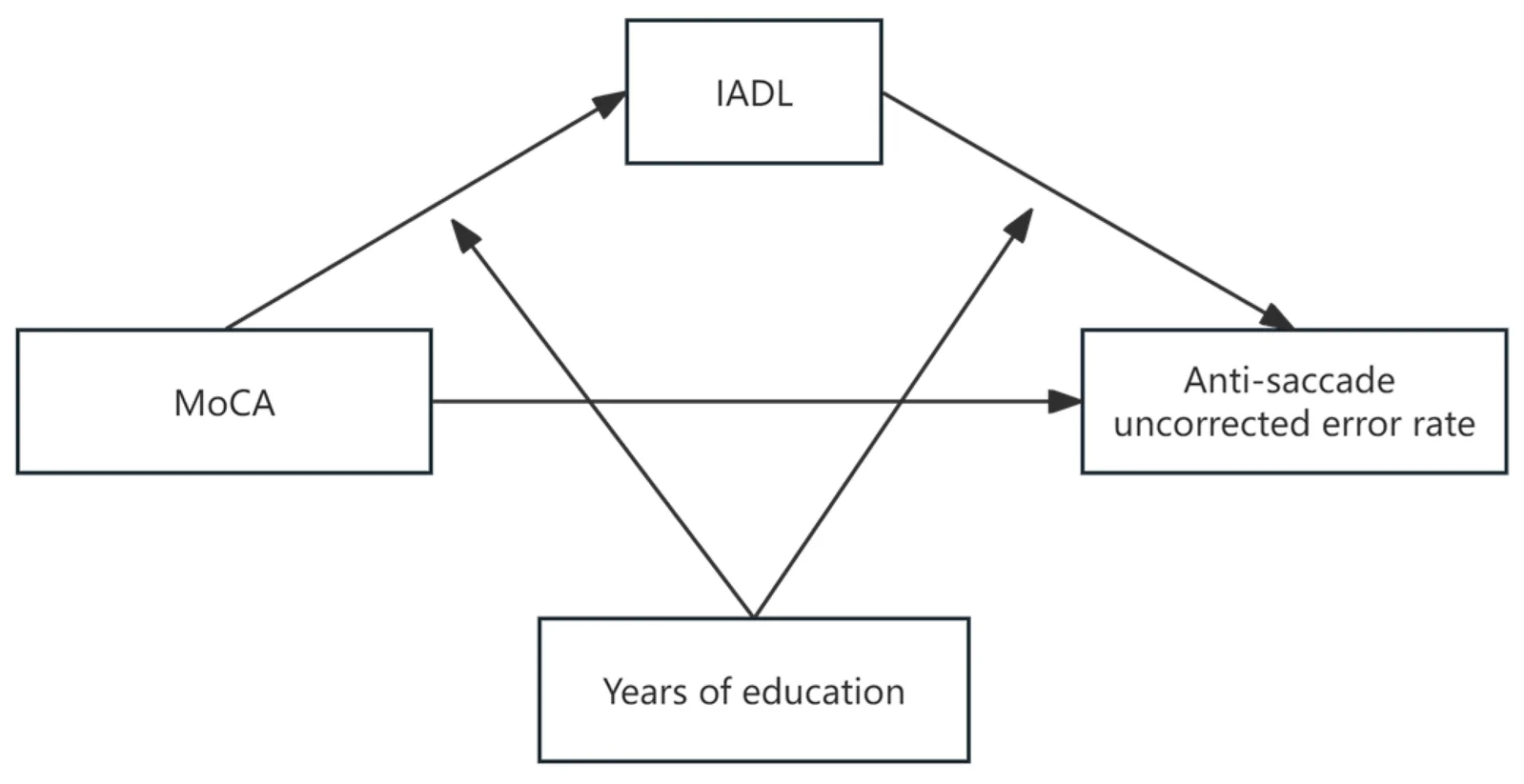
Conceptual framework of the moderated mediation effect. Note: The moderation effect of the first path (MoCA → IADL) was tested separately by PROCESS Model 7; the moderation effect of the latter path (IADL → anti-saccade uncorrected error rate) was tested separately by PROCESS Model 14. The two moderation paths were not estimated simultaneously in the same model. MoCA, Montreal Cognitive Assessment; IADL, Instrumental Activities of Daily Living.

Mediation effect testing used PROCESS 4.2 Model 4, with MoCA as the independent variable, IADL as the mediator, and anti-saccade uncorrected error rate, corrected error rate, and correct rate as dependent variables, respectively, with years of education, age, and vascular risk factors as covariates. The conceptual framework of the moderated mediation analysis model is shown in Figure 1, using PROCESS 4.2 Model 7 (testing whether education moderates the first path “MoCA → IADL”) and Model 14 (testing whether education moderates the latter path “IADL → oculomotor indicator”), with years of education as the moderator and age and vascular risk factors as covariates. All three oculomotor outcomes used the same model specification described above. In Model 7 and Model 14, years of education was included as a moderator in the interaction term and no longer simultaneously as an ordinary covariate. All continuous variables were mean-centered before constructing interaction terms. The significance of mediation effects and moderated mediation effects was calculated using the Bootstrap method (5000 resamples) with 95% confidence intervals; if the confidence interval did not include 0, it was considered statistically significant. Simple slope analysis was used to decompose significant interaction effects, and the Johnson-Neyman method was used to determine the significance threshold of the moderator. All tests were two-sided.

It should be particularly noted that a significant IADL × years of education interaction term in Model 14 and the moderated mediation index test different hypotheses. The former addresses whether the conditional association of the latter path “IADL → oculomotor indicator” varies with years of education; the latter addresses whether the indirect effect itself varies with years of education. Therefore, this study reported the path interaction term and the moderated mediation index separately, and did not infer that moderated mediation is established based on a significant path interaction term. If the confidence interval of the moderated mediation index included 0, it was clearly reported that the moderating effect of years of education on the overall mediation effect was not statistically supported.

Finally, to address multiplicity issues arising from screening of the three oculomotor outcomes and subsequent interaction testing, this study used Benjamini–Hochberg FDR correction. All indirect association and conditional association results were interpreted as cross-sectional associations, without causal inference. Test family 1 was “outcome screening” multiplicity, including the significance tests of indirect effects for the three outcomes (m = 3, α = 0.05); test family 2 was “interaction testing” multiplicity, including the IADL × years of education interaction terms for the three outcomes in Model 14 (m = 3, α = 0.05). After BH correction, *p* values were calculated as *p* × (m/i) and subjected to monotonicity adjustment. Those still significant after correction were regarded as robust findings, and those no longer significant after correction were reported as exploratory trends and not as main conclusions. In addition, this study did not conduct formal direct comparisons of indirect effects among the three anti-saccade outcomes; therefore, neither the Results nor the Discussion interpret “one outcome significant, another not significant” as evidence of outcome-specific mediation or different neural mechanisms.

## 3. Results

### 3.1. Basic Characteristics of Participants

A total of 171 patients with cerebral small vessel disease (CSVD) were included in this study. Demographic and clinical characteristics are shown in Table 1: mean age was 67.92 ± 6.74 years, and mean years of education was 11.16 ± 3.13 years. In terms of scale assessments, the mean MoCA score was 24.08 ± 3.53, indicating overall cognition in the mildly impaired range; the IADL score was 23.29 ± 0.93, indicating that patients’ daily instrumental activity ability was generally fair. The observed range of IADL (19–24), median (23), interquartile range (1), and proportion with the highest score (24 points) (49.7%) indicated a significant ceiling effect. In terms of emotional status, HAMD and HAMA scale scores were 4.73 ± 3.85 and 10.41 ± 5.51, respectively, suggesting that the overall emotional state of the sample was stable/mildly abnormal. In the anti-saccade task, the uncorrected error rate, corrected error rate, and correct rate were 13.17 ± 21.71%, 78.34 ± 31.04%, and 45.71 ± 30.25%, respectively, with large inter-individual differences. Among these, the uncorrected error rate and correct rate were calculated based on valid anti-saccade trials from all 171 patients; the corrected error rate used initially directionally erroneous trials as the conditional denominator, and data were calculable for 163 of the 171 patients, with 8 patients treated as missing values because they had no initially directionally erroneous trials. Because the denominators of the three metrics differ, their percentages do not necessarily sum to 100%.

### 3.2. Correlation Analysis Among Core Variables

Pearson correlation analysis described the correlations of the three anti-saccade indicators with MoCA and IADL (Table 2). The results showed that anti-saccade uncorrected error rate was significantly correlated with MoCA (r = −0.275, *p* < 0.01) and IADL (r = −0.373, *p* < 0.01); anti-saccade corrected error rate was correlated with MoCA (r = 0.192, *p* < 0.05) and IADL (r = 0.308, *p* < 0.01); anti-saccade correct rate was correlated with MoCA (r = 0.282, *p* < 0.01) and IADL (r = 0.210, *p* < 0.01).

### 3.3. Mediating Effect of IADL Between MoCA and Anti-Saccade Uncorrected Error Rate

In the mediation analysis of this section, when the dependent variable was the anti-saccade uncorrected error rate, its denominator was the number of valid anti-saccade trials, and the number of patients for whom it could be calculated was 171. Model 4 of PROCESS 4.2 was used to test the mediating effect of IADL between MoCA and the oculomotor metric (Table 3), with MoCA as the independent variable, IADL as the mediator, the anti-saccade uncorrected error rate as the dependent variable, and age, years of education, and vascular risk factors (hypertension, diabetes, and hyperlipidemia) as covariates.

The results showed that the total effect of MoCA on the anti-saccade uncorrected error rate was significant (*p* = 0.002, 95% CI [−2.474, −0.568]); the predictive effect of MoCA on IADL was significant (*p* = 0.016, 95% CI [0.009, 0.089]); after controlling for MoCA, the predictive effect of IADL on the anti-saccade uncorrected error rate was significant (*p* < 0.001, 95% CI [−12.138, −5.199]). After controlling for IADL, the direct effect of MoCA on the anti-saccade uncorrected error rate remained significant (*p* = 0.018, 95% CI [−2.004, −0.188]). Bootstrap results with 5000 resamples showed that the indirect effect of IADL between MoCA and the anti-saccade uncorrected error rate was significant (95% BootCI [−1.138, −0.003]).

The results suggest that this pattern is consistent with the indirect association hypothesis that “lower MoCA may be associated with a higher anti-saccade uncorrected error rate through impaired IADL”; however, causal mediation cannot be inferred on this basis.

### 3.4. Moderation Effect of Years of Education in the Mediation Model Related to Anti-Saccade Uncorrected Error Rate

PROCESS 4.2 Model 7 and Model 14 were used to test the moderating effects of years of education on the first half-pathway (MoCA → IADL) and the second half-pathway (IADL → anti-saccade uncorrected error rate) of the mediation model, respectively (Table 4), with years of education as the moderator and age and vascular risk factors (hypertension, diabetes, and hyperlipidemia) as covariates.

**Table 4:** Test of the moderation effect of years of education in the moderated mediation model (PROCESS Model 7 & Model 14).

Model and Path	Dependent Variable	Predictor	coeff	SE	t	*p*	95% CI	R^2^-Change
Model 7	IADL	MoCA × years of education	0.002	0.006	0.389	0.698	[−0.009, 0.013]	0.001
Model 14	Anti-saccade uncorrected error rate	IADL × years of education	1.060	0.496	2.139	0.034 *	[0.081, 2.039]	0.022
Index of moderated mediation (Index)	—	Years of education	Model 7: −0.018	0.074 ^a^	—	—	[−0.183, 0.119] ^b^	—
Index of moderated mediation (Index)	—	Years of education	Model 14: 0.044	0.036 ^a^	—	—	[−0.021, 0.123] ^b^	—

Note: * *p* < 0.05. In Model 7, MoCA and years of education were mean-centered; in Model 14, IADL and years of education were mean-centered. The number of Bootstrap resamples was 5000. ^a^ denotes the BootSE; ^b^ denotes the BootCI; if the confidence interval includes 0, the effect is considered non-significant. The anti-saccade uncorrected error rate was based on data from 171 patients. Although the interaction term in Model 14 was significant (*p* = 0.034), the R^2^-change was only 0.022, indicating that the actual explanatory power of the moderating effect was limited and should be interpreted with caution. The moderated mediation index was not obtained by directly multiplying the path a coefficient from the simple mediation model in Table 3 by the interaction term coefficient in Table 5, but was jointly calculated from the path coefficients and moderation coefficients within the same moderated mediation model (Model 14). Table 3 and Table 5 are derived from different model specifications, and their coefficients should not be directly multiplied across models. The significant IADL × years of education interaction term in Model 14 only suggests that the conditional association of the second half-pathway may vary with years of education; it does not indicate that moderated mediation was statistically supported. In this study, the 95% BootCI of the moderated mediation index in Model 14 included 0.

**Table 5:** Results of conditional effects across different education groups.

Years of Education Group	Evaluation Point (M ± 1 SD)	Effect	SE	t	*p*	95%BootCI
Low education group	M − 1 SD (8.03 years)	−12.862	2.620	−4.909	<0.001 **	[−18.036, −7.688]
Medium education group	Mean (11.16 years)	−9.541	1.785	−5.345	<0.001 **	[−13.067, −6.016]
High education group	M + 1 SD (14.29 years)	−6.220	2.081	−2.989	0.003 **	[−10.330, −2.111]

Note: ** *p* < 0.01. The number of Bootstrap resamples was 5000. The effect size represents the absolute percentage-point change in the anti-saccade uncorrected error rate for each 1-point increase in IADL; a larger absolute value of the negative effect indicates a stronger harmful effect of impaired IADL on oculomotor inhibition. Years of education were mean-centered in the model, and the evaluation points in the table have been converted back to actual years of education. Johnson-Neyman analysis showed that when years of education exceeded 15.55 years, the 95% Bootstrap confidence interval of the conditional effect included 0. It should be noted that this only indicates that the conditional association is no longer statistically significant at this educational level, and does not prove that the association completely disappears or that a clinical protective threshold exists. The anti-saccade uncorrected error rate was based on data from 171 patients.

The results showed that in Model 7, the MoCA × years of education interaction term did not significantly predict IADL (*p* = 0.698, 95% CI [−0.009, 0.013]); in Model 14, the IADL × years of education interaction term significantly predicted the anti-saccade uncorrected error rate (*p* = 0.034, 95% CI [0.081, 2.039]). Bootstrap results with 5000 resamples showed that the moderated mediation index was −0.018 for Model 7 (95% BootCI [−0.183, 0.119]) and 0.044 for Model 14 (95% BootCI [−0.021, 0.123]).

Two types of results need to be clearly distinguished: the significant IADL × years of education interaction term in Model 14 indicates that years of education may moderate the conditional association of the second half-pathway, “IADL → anti-saccade uncorrected error rate”; whereas the moderated mediation index tests a different hypothesis, namely whether the indirect effect varies with years of education. In this study, the confidence intervals of the moderated mediation indices in both models included 0, suggesting that the moderating effect of years of education on the overall mediation effect was not statistically supported. Therefore, a significant pathway interaction term cannot be equated with the establishment of moderated mediation. Taken together, although the pathway interaction term in Model 14 was significant, its incremental variance was limited, and the overall moderated mediation index was not significant, further supporting that the moderating effect of years of education should be cautiously interpreted as an exploratory trend rather than a robust moderating effect.

### 3.5. Conditional Effects of IADL on the Anti-Saccade Uncorrected Error Rate Across Different Levels of Years of Education

To further elucidate the moderating effect of years of education, simple slope analysis was used to examine the conditional effects of IADL on the oculomotor metric across different levels of years of education (Table 5).

The results showed that as years of education increased, the absolute value of the negative effect of IADL on the anti-saccade uncorrected error rate gradually decreased: the effect was −12.862 in the low education group (M − 1 SD, 8.03 years; *p* < 0.001, 95% BootCI [−18.036, −7.688]); −9.541 in the medium education group (mean, 11.16 years; *p* < 0.001, 95% BootCI [−13.067, −6.016]); and −6.220 in the high education group (M + 1 SD, 14.29 years; *p* = 0.003, 95% BootCI [−10.330, −2.111]). The Johnson-Neyman method showed that when years of education reached 15.55 years, the 95% confidence interval of the conditional effect of IADL on the anti-saccade uncorrected error rate included 0 for the first time (*p* ≥ 0.05).

It should be noted that the Johnson-Neyman boundary is only used to identify the region where the confidence interval of the conditional association includes zero, and does not mean that the effect completely disappears or that there is an absolute protective educational threshold. In the present sample, only 12 participants (approximately 7% of the total sample) had years of education exceeding this critical value, representing a relatively limited sample size; therefore, this value should not be interpreted as a clinical intervention threshold. This analysis addressed only the conditional effect of the second half-pathway and cannot serve as evidence that moderated mediation was significant; the 95% BootCI of the moderated mediation index included 0.

### 3.6. Regression Diagnostics and HC3 Robust Standard Error Sensitivity Analysis

Because the anti-saccade uncorrected error rate is a percentage metric (0–100%), with an observed range of 0–100, a median of 0, an interquartile range of 20, a zero-value proportion of 56.7%, and a maximum-score proportion of 1.7%, its distribution exhibited clear zero inflation and right skew. Therefore, this study further performed regression diagnostics for the mediation model (residual diagnostic plots are shown in the Supplementary Materials (Figures S1–S3) and conducted sensitivity analyses using HC3 heteroskedasticity-robust standard errors.

(1) The mediation effect was re-evaluated using HC3 heteroskedasticity-robust standard errors (Table 6): the results showed that the total effect of MoCA on the anti-saccade uncorrected error rate remained significant (*p* = 0.012, 95% CI [−2.701, −0.341]). The predictive effect of MoCA on IADL was not significant (path a: *p* = 0.077, 95% CI [−0.005, 0.103]); the negative predictive effect of IADL on the anti-saccade uncorrected error rate remained significant (path b: *p* = 0.006, 95% CI [−14.851, −2.486]). After controlling for IADL, the direct effect of MoCA on the anti-saccade uncorrected error rate remained significant (c′: *p* = 0.028, 95% CI [−2.071, −0.121]). Bootstrap test results showed that the indirect effect of MoCA on the anti-saccade uncorrected error rate through IADL remained significant (95% BootCI [−1.132, −0.002]), with the confidence interval not including 0.

(2) HC3 robust standard errors were used to test the moderated mediation effect in Model 14 (Table 7): the results showed that the interaction term between IADL and years of education did not significantly predict the anti-saccade uncorrected error rate (*p* = 0.211, 95% CI [−0.605, 2.726]), indicating that years of education did not have a significant moderating effect on the second half-pathway, IADL → anti-saccade uncorrected error rate. Further testing of the moderated mediation index showed a 95% BootCI of [−0.021, 0.124], with the confidence interval including 0.

In summary, these findings suggest that under HC3 robust standard errors, the confidence interval of the core path b still did not include 0, path a was no longer significant (*p* = 0.077), and the upper limit of the Bootstrap confidence interval for the indirect effect was close to 0. In addition, the HC3-based Model 14 test showed that the interaction term between IADL and years of education no longer significantly predicted the anti-saccade uncorrected error rate (*p* = 0.211), and the confidence interval of the moderated mediation index included 0. Taken together, this indirect association showed only limited robustness and was accompanied by considerable parameter uncertainty; the moderating effect of years of education on the IADL → anti-saccade uncorrected error rate pathway was not supported.

### 3.7. Multiplicity Correction Results

To address multiplicity issues arising from screening of the three oculomotor outcomes and subsequent interaction testing, Benjamini–Hochberg FDR correction was used. It should be noted that the bootstrap *p* values in the tables were calculated as the ratio of the indirect effect to its bootstrap standard error under a normal approximation and serve only as an exploratory reference; interval inference for the indirect effects relied primarily on percentile bootstrap confidence intervals, which do not depend on the normality assumption. The overall conclusions after multiplicity correction do not depend on this normal-approximation *p* value.

(1) Benjamini–Hochberg FDR correction (Table 8): The results showed that none of the indirect effects for the three antisaccade outcomes reached the conventional level of statistical significance (uncorrected error rate: Bootstrap *p* = 0.153, BH-adjusted *p* = 0.212; corrected error rate: Bootstrap *p* = 0.168, BH-adjusted *p* = 0.212; correct rate: Bootstrap *p* = 0.212, BH-adjusted *p* = 0.212). It should be noted that the unadjusted bootstrap *p* values were already greater than 0.05; therefore, their non-significance was not solely attributable to FDR multiplicity correction. Given that the bootstrap *p* values are only exploratory references based on the ratio of the indirect effect to its standard error under a normal approximation, we further assessed robustness using bootstrap confidence intervals. Taken together with the multiplicity correction framework and the bootstrap test results, no indirect effect for any antisaccade outcome was found to be statistically robust. The above indirect effects should be interpreted cautiously as exploratory trends, and their confirmation requires further validation in larger samples.

(2) FDR correction for moderated mediation effects (Table 9): FDR correction was performed on the IADL × years of education interaction terms for the three outcomes in Model 14 (test family “interaction testing,” m = 3, α = 0.05). The results showed that after correction, the interaction term of uncorrected error rate was no longer significant (BH-corrected *p* = 0.051), only the interaction term of anti-saccade corrected error rate remained significant (BH-corrected *p* = 0.048), and the interaction term of correct rate was also not significant (BH-corrected *p* = 0.384).

## 4. Discussion

This study explored the mediating association of “global cognition (MoCA) → instrumental activities of daily living (IADL) → prefrontal inhibitory control (anti-saccade uncorrected error rate)” in patients with CSVD, and tested the moderating role of years of education. There were three core findings: (1) the indirect association of IADL between MoCA and the anti-saccade uncorrected error rate was significant before correction but was no longer significant after BH-FDR correction (Bootstrap *p* = 0.153, BH-corrected *p* = 0.212); it should be noted that the unadjusted bootstrap *p* value was already greater than 0.05, so its non-significance was not solely attributable to FDR multiplicity correction. Given that the bootstrap *p* value is based only on a normal approximation and serves as an exploratory reference, this study primarily relied on percentile bootstrap confidence intervals, which do not depend on the normality assumption, for inference, and the indirect effect was judged overall not to reach statistical robustness; (2) the indirect effects for all three anti-saccade outcomes were no longer significant after BH-FDR correction (all BH-corrected *p* = 0.212), and this study did not perform formal direct comparisons of the indirect effects among outcomes, so outcome-specific mediation or distinct neural mechanisms cannot be inferred on this basis; (3) the interaction term of years of education on the second half-pathway, “IADL → anti-saccade uncorrected error rate,” was significant before correction (*p* = 0.034) but was no longer significant after BH-FDR correction (BH-corrected *p* = 0.051); more importantly, the moderated mediation indices for the uncorrected error rate, corrected error rate, and correct rate were all non-significant, suggesting that the moderated mediation hypothesis that “the indirect effect varies with years of education” was not statistically supported, and therefore this study cannot claim that years of education moderated the overall mediation effect. All the above results are based on an exploratory cross-sectional analysis, and the selection of the primary oculomotor outcome was also exploratory; temporal or causal sequences cannot be determined, reverse associations and common causes remain plausible explanations, and the relevant findings still require further validation in independent samples and longitudinal designs.

### 4.1. Mediating Role of IADL

In this study, the indirect association of IADL between MoCA and the anti-saccade uncorrected error rate was significant before correction but was no longer significant after BH-FDR correction (Bootstrap *p* = 0.153, BH-corrected *p* = 0.212). It should be noted that the unadjusted bootstrap *p* value was already greater than 0.05, indicating that its non-significance was not solely attributable to FDR multiplicity correction; moreover, this *p* value was based on a normal approximation and serves only as an exploratory reference, with interval inference for the indirect effect relying primarily on percentile bootstrap confidence intervals. Moreover, in the HC3 robust standard error sensitivity analysis, path a in Model 4 of the uncorrected error rate model was no longer significant (*p* = 0.077), the upper limit of the Bootstrap confidence interval for the indirect effect was close to 0, and the IADL × years of education interaction term in Model 14 was also no longer significant (*p* = 0.211), suggesting that this indirect association showed only limited robustness and was accompanied by considerable parameter uncertainty. This indicates that, in the specified cross-sectional model, there was a direct association between cognitive function and oculomotor inhibitory ability in patients with CSVD, as well as an estimated indirect association via IADL; however, this does not demonstrate that cognitive decline causally aggravates prefrontal inhibitory control impairment through IADL. One possible explanation is that complex daily activities (such as financial management and medication management) require substantial executive resources, and when CSVD leads to cognitive decline, the remaining cognitive resources are insufficient to effectively inhibit prepotent reflexive saccades [17,18]; however, this mechanism remains hypothetical and requires further testing in longitudinal studies. From a neural mechanism perspective, both the effective performance of IADL and anti-saccade inhibition depend on the integrity of the frontal-striatal circuitry, especially the connectivity between the dorsolateral prefrontal cortex and the caudate nucleus [24,42]. CSVD-related white matter hyperintensities can disrupt this network [43,44] and may be associated with simultaneous impairment of daily problem-solving ability and voluntary oculomotor control. Therefore, within a cross-sectional association framework, IADL may amplify the association between cognitive deficits and oculomotor inhibitory function—mild cognitive deficits are amplified through IADL decline and ultimately manifest as significantly impaired oculomotor inhibitory function. This finding is consistent with the synergistic model of Jokinen et al. [18], and this study further extends this synergistic effect to the oculomotor domain, suggesting that IADL is not only a consequence of cognitive impairment but may also be related to the association pathway of executive dysfunction. However, it should be noted that path a was no longer significant under HC3 robust standard errors, and this study did not perform formal direct comparisons of the indirect effects among the three anti-saccade outcomes; therefore, the significance for the uncorrected error rate and non-significance for the corrected error rate and correct rate cannot be interpreted as outcome-specific mediation.

### 4.2. Interpretation of the Anti-Saccade Uncorrected Error Rate Results

Before multiplicity correction, the mediation effect was significant only for the anti-saccade uncorrected error rate, whereas the corrected error rate and correct rate showed no significant indirect effects; after BH-FDR correction, the indirect effects for all three anti-saccade outcomes were no longer significant (all BH-corrected *p* = 0.212). It should be noted that the unadjusted bootstrap *p* values were already greater than 0.05; therefore, this non-significant result did not arise solely from FDR multiplicity correction. At the same time, the bootstrap *p* values are based on a normal approximation and serve only as an exploratory reference, with robustness assessment of the indirect effects relying primarily on percentile bootstrap confidence intervals. However, it must be emphasized that a significant result for one outcome and a non-significant result for another cannot establish that the indirect effects differ between the two. This study did not perform formal direct comparisons of the indirect effects among the three outcomes, nor did it specifically test the dependence among outcomes. Therefore, the above results should not be interpreted as evidence of outcome-specific mediation or distinct neural mechanisms.

From the perspective of metric definitions, the three anti-saccade metrics have different denominators, directions, and psychometric meanings: the uncorrected error rate is based on all valid trials and reflects inhibitory failure; the corrected error rate uses initially directionally erroneous trials as the conditional denominator and reflects the ability to correct after an error; and the correct rate is based on all valid trials and reflects the ultimate success of inhibition. The three are not simple repeated measures of the same latent variable, and their indirect effect estimates should not be directly compared across metrics. The uncorrected error rate mainly reflects the functional integrity of the “direct inhibitory pathway” (dorsolateral prefrontal cortex → frontal eye field → substantia nigra pars reticulata), whereas the corrected error rate depends on the error monitoring and correction circuit formed by the anterior cingulate cortex and pre-supplementary motor area [20,22]. The pathological changes of CSVD mainly involve frontal white matter fibers connecting the dorsolateral prefrontal cortex and subcortical structures, whereas anterior cingulate-related circuits may remain relatively intact or possess some compensatory capacity in the early stage of the disease [7]. These mechanistic explanations can serve as hypotheses for future research, but under the current cross-sectional design and in the absence of direct comparisons among outcomes, they cannot serve as evidence of outcome-specific mediation. Therefore, a more cautious statement is: the anti-saccade uncorrected error rate, as a behavioral marker of CSVD-related prefrontal disinhibition, warrants further validation of its targeting and sensitivity in independent samples; its causal significance and its differences from the corrected error rate and correct rate still require dedicated designs for testing.

### 4.3. Path Specificity of Education Moderation

Education did not significantly moderate the first half-pathway, “MoCA → IADL” (interaction term *p* = 0.698). In Model 14, the moderating effect of education on the second half-pathway, “IADL → uncorrected error rate,” was significant before correction (interaction term *p* = 0.034) but was no longer significant after BH-FDR correction (BH-corrected *p* = 0.051); in contrast, the moderating effect of education on the “IADL → corrected error rate” pathway was significant after BH-FDR correction (BH-corrected *p* = 0.048). However, a distinction must be made between the second half-pathway interaction term and the moderated mediation index: the former only indicates that the conditional association of “IADL → anti-saccade uncorrected error rate” may vary with years of education, whereas the latter tests whether the indirect effect itself varies with years of education. In this study, the moderated mediation indices for the uncorrected error rate, corrected error rate, and correct rate were all non-significant, suggesting that the moderated mediation hypothesis was not statistically supported. Therefore, even if a moderating effect of education exists, it is mainly manifested at the level of conditional association of a specific pathway rather than at the level of the overall indirect effect; after multiplicity correction, this finding shifted between the uncorrected error rate and the corrected error rate, suggesting that it is not entirely robust and requires further validation in larger samples. These moderation results describe cross-sectional conditional associations and cannot demonstrate that education changed the causal effect of IADL on oculomotor function.

Simple slope analysis showed that the effect of IADL on the uncorrected error rate was strongest in the low education group (*p* < 0.001), and this effect was significantly attenuated in the high education group (*p* = 0.003); moreover, the Johnson-Neyman method defined that when years of education exceeded 15.55 years, the effect of IADL on the uncorrected error rate was no longer statistically significant (*p* ≥ 0.05). However, this analysis addressed only the conditional effect of the second half-pathway, and the interaction term was no longer significant after BH-FDR correction (BH-corrected *p* = 0.051), with the moderated mediation index also non-significant. Therefore, these results should be regarded as exploratory conditional associations [45,46] and cannot be interpreted as indicating that highly educated individuals altered the mediation pathway through neural compensatory strategies, nor can they serve as evidence that education moderates the overall mediation effect. This finding is complementary to the traditional view of Jokinen et al. [33] and Li et al. [34] that education mainly protects cognition against brain pathology—future longitudinal designs could further test whether education acts at the “behavior-execution” interface.

### 4.4. Clinical and Translational Implications

The results of this study have multiple practical implications. First, the “uncorrected error rate” measured by a brief anti-saccade task may have some applicability for rapid screening of prefrontal dysfunction in clinical patients with CSVD, but the clinical value of this metric still requires further validation in independent samples. Second, intervention strategies should not be limited to cognitive training; maintaining or even improving IADL through occupational therapy or assistive technology may have positive effects on cognitive intervention. Third, the conditional association between years of education and the second half-pathway suggests that educational attainment may be associated with the relationship between IADL and executive function, but this moderating effect was no longer significant after multiplicity correction, and the moderated mediation index was also non-significant; therefore, it can only serve as an exploratory hint rather than an established basis for intervention thresholds.

In clinical interpretation, it should be noted that the selection of the primary oculomotor outcome in this study was an exploratory analysis; although the three anti-saccade metrics used the same model specification and were uniformly subjected to BH-FDR correction, given that the unadjusted bootstrap *p* values were themselves non-significant and that the bootstrap *p* values are based only on a normal approximation and serve as an exploratory reference, this study assessed robustness comprehensively using bootstrap confidence intervals and confirmed that the indirect effects for all three outcomes were no longer significant after correction; moreover, this study did not perform formal direct comparisons of the indirect effects among the three outcomes, so “significant for the uncorrected error rate and non-significant for the corrected error rate and correct rate” cannot be interpreted as outcome-specific mediation. Regarding educational moderation, the pathway interaction term and the moderated mediation index in Model 14 address different hypotheses; in this study, the moderated mediation indices for all outcomes were not statistically supported. Therefore, the above clinical implications should all be treated as exploratory associations and require validation in longitudinal and interventional studies.

### 4.5. Limitations and Future Directions

This study has several limitations. The cross-sectional design cannot infer causality and requires longitudinal follow-up for validation. First, the functional level of the study participants was relatively high, IADL scores showed limited variability and a significant ceiling effect (49.7% achieved the maximum score), which may weaken the observed associations and limit the generalizability of the results to populations with more severe functional impairment. Years of education as the sole proxy indicator of cognitive reserve is overly simplistic; future studies should incorporate multidimensional reserve indicators such as occupational attainment and leisure intellectual activities. Anxiety/depressive symptoms may partially explain or mediate the associations, and may also reduce statistical power by increasing the number of covariates; however, the conclusions of this study are limited to cross-sectional descriptive associations, cannot exclude residual confounding by emotional symptoms, and do not involve causal inference. This study did not collect imaging indicators such as WMH volume or fractional anisotropy; future studies could construct a multilevel mediation model of “brain structural damage → cognition → IADL → oculomotor behavior” to rigorously test the hypotheses of this study.

In addition, before correction, the mediation effect was significant for the uncorrected error rate but not for the corrected error rate, whereas after FDR correction none of the three were significant; this may be related to the fact that this cohort was in the mild-to-moderate impairment stage, and may also be related to differences among the three metrics in denominators, directions, and psychometric properties. However, because no direct comparisons among outcomes were performed, outcome-specific mechanisms cannot be inferred on this basis. Moreover, the bootstrap *p* values for the indirect effects were calculated based on a normal approximation; if the bootstrap sampling distribution deviates from normality, these *p* values should be interpreted with caution. Future studies could use Bayesian mediation analysis or larger samples to further verify the robustness of the indirect effects. In more advanced CSVD or under conditions of higher task difficulty, the error monitoring circuit may also become involved; future studies could employ anti-saccade paradigms with multiple difficulty gradients. The corrected error rate uses initially directionally erroneous trials as the conditional denominator; in this study, 8 patients could not have this metric calculated because they had no initially directionally erroneous trials. Future studies could expand the sample size or add difficulty gradients in the task design to improve the calculability and stability of the corrected error rate metric.

Finally, the selection of the primary oculomotor outcome in this study was not a pre-specified confirmatory hypothesis, but rather an exploratory choice combining theoretical background and preliminary statistical results; the results for the three anti-saccade metrics were all no longer significant after multiplicity correction, and no direct comparisons of the indirect effects among outcomes were performed; regarding educational moderation, the pathway interaction term and the moderated mediation index in Model 14 also address different hypotheses. Therefore, the relevant mediation and moderation conclusions all require further validation in independent samples and longitudinal designs.

## 5. Conclusions

In summary, this study suggests that, before correction, IADL may partially mediate the association between global cognition and the anti-saccade uncorrected error rate in patients with CSVD, but this indirect effect was not statistically supported after FDR correction; it should be noted that the unadjusted bootstrap *p* value was already greater than 0.05, and this *p* value is based on a normal approximation and serves only as an exploratory reference; inference regarding the indirect effect relied primarily on bootstrap confidence intervals, and the indirect effect was judged overall not to reach statistical robustness; moreover, the indirect effects among outcomes were not formally directly compared, so outcome-specific mediation cannot be inferred on this basis. The moderating effect of years of education on the overall mediation effect was not statistically supported: the Model 14 interaction term was no longer significant after FDR correction, and the confidence intervals of the moderated mediation indices all included 0. All the above results are cross-sectional exploratory findings, cannot infer causality, and require validation in independent samples and longitudinal designs.

## Figures and Tables

**Table 1:** Demographic and clinical characteristics of patients with CSVD (N = 171).

Variable	Mean ± SD/Proportion
Age (years)	67.92 ± 6.74
Sex (male/female)	46/125
Years of education (years)	11.16 ± 3.13
MoCA (score)	24.08 ± 3.53
IADL (score)	23.29 ± 0.93
HAMD (score)	4.73 ± 3.85
HAMA (score)	10.41 ± 5.51
Anti-saccade uncorrected error rate (%)	13.17 ± 21.71
Anti-saccade corrected error rate (%)	78.34 ± 31.04
Anti-saccade correct rate (%)	45.71 ± 30.25

Note: MoCA, Montreal Cognitive Assessment; IADL, Instrumental Activities of Daily Living; HAMD, Hamilton Depression Scale; HAMA, Hamilton Anxiety Scale.

**Table 2:** Correlation analysis of MoCA, IADL, and anti-saccade uncorrected error rate in patients with CSVD.

Dependent Variable	Correlation with MoCA (r)	Correlation with IADL (r)
Anti-saccade uncorrected error rate	−0.275 **	−0.373 **
Anti-saccade corrected error rate	0.192 *	0.308 **
Anti-saccade correct rate	0.282 **	0.210 **

Note: * *p* < 0.05, ** *p* < 0.01. MoCA, Montreal Cognitive Assessment. Data are median; IADL, Instrumental Activities of Daily Living.

**Table 3:** Regression analysis of the mediating effect of IADL between MoCA and anti-saccade uncorrected error rate.

Effect Type	Path	Effect	SE	t	*p*	95% CI
Total effect	MoCA → anti-saccade uncorrected error rate	−1.521	0.483	−3.151	0.002 **	[−2.474, −0.568]
Path a	MoCA → IADL	0.049	0.020	2.440	0.016 *	[0.009, 0.089]
Path b	IADL → anti-saccade uncorrected error rate	−8.668	1.757	−4.934	<0.001 **	[−12.138, −5.199]
Direct effect (c’)	MoCA → anti-saccade uncorrected error rate (after controlling for IADL)	−1.096	0.460	−2.384	0.018 *	[−2.004, −0.188]
Indirect effect (a × b)	MoCA → IADL → anti-saccade uncorrected error rate	−0.425	0.299 ^#^	—	—	[−1.138, −0.003] ^#^

Note: * *p* < 0.05, ** *p* < 0.01. ^#^ The indirect effect was estimated using Bootstrap with 5000 resamples, BootSE = 0.299, 95% BootCI = [−1.138, −0.003]. The total effect, direct effect, path a, and path b were all derived from the Model 4 output. The anti-saccade uncorrected error rate was based on data from 171 patients.

**Table 6:** Regression analysis of the mediating effect of IADL between MoCA and anti-saccade uncorrected error rate (HC3 robust standard errors).

Effect Type	Path	Effect	SE	t	*p*	95% CI
Total effect	MoCA → anti-saccade uncorrected error rate	−1.521	0.598	−2.546	0.012 *	[−2.701, −0.341]
Path a	MoCA → IADL	0.049	0.028	1.777	0.077	[−0.005, 0.103]
Path b	IADL → anti-saccade uncorrected error rate	−8.668	3.131	−2.769	0.006 **	[−14.851, −2.486]
Direct effect (c’)	MoCA → anti-saccade uncorrected error rate (after controlling for IADL)	−1.096	0.494	−2.220	0.028 *	[−2.071, −0.121]
Indirect effect (a × b)	MoCA → IADL → anti-saccade uncorrected error rate	−0.425	0.298 ^#^	—	—	[−1.132, −0.002] ^#^

Note: * *p* < 0.05, ** *p* < 0.01. All regression coefficients were estimated based on HC3 heteroskedasticity-robust standard errors; ^#^ The indirect effect was estimated using Bootstrap with 5000 resamples, BootSE = 0.298, 95% BootCI = [−1.132, −0.002]. The anti-saccade uncorrected error rate was based on data from 171 patients.

**Table 7:** Test of the Moderating Effect of Years of Education in the Moderated Mediation Model (HC3 Robust Standard Errors).

Model and Path	Dependent Variable	Predictor	coeff	SE	t	*p*	95% CI	R^2^-Change
Model 14	Anti-saccade uncorrected error rate	IADL × years of education	1.060	0.843	1.257	0.211	[−0.605, 2.726]	0.022
Index of moderated mediation (Index)	—	Years of education	0.044	0.037 ^a^	—	—	[−0.021, 0.124] ^b^	—

Note: All regression coefficients were estimated based on HC3 heteroskedasticity-robust standard errors; The number of Bootstrap resamples was 5000. ^a^ denotes the BootSE; ^b^ denotes the BootCI; if the confidence interval includes 0, the effect is considered non-significant. The anti-saccade uncorrected error rate was based on data from 171 patients.

**Table 8:** FDR Correction Results for the Indirect Effects of the Three Anti-Saccade Outcomes.

Outcome	Indirect Effect	Effect	BootSE	Bootstrap *p*	BH-Corrected *p*	Significant After Correction
Uncorrected error rate	a × b	−0.425	0.299	0.153	0.212	No
Corrected error rate	a × b	0.488	0.354	0.168	0.212	No
Correct rate	a × b	0.242	0.194	0.212	0.212	No

Note: The indirect effects were tested using the percentile Bootstrap method with 5000 resamples. Bootstrap *p* values were calculated based on the ratio of the indirect effect to its Bootstrap standard error under a normal approximation; the three *p* values were then subjected to Benjamini–Hochberg FDR correction (test family m = 3, α = 0.05). After correction, none of the three indirect effects remained significant (all BH-corrected *p* = 0.212). Bootstrap confidence intervals are percentile-based results and were used only for inference regarding individual indirect effects; they did not participate in multiplicity correction.

**Table 9:** FDR correction results for the latter-path interaction terms of the three anti-saccade outcomes in Model 14.

Outcome	Indirect Effect	Raw *p* Value	BH-Corrected *p* Value	Significance After Correction
Uncorrected error rate	IADL × education	0.034 *	0.051	No
Corrected error rate	IADL × education	0.016 *	0.048	Yes
Correct rate	IADL × education	0.384	0.384	No

Note: The test family was the multiplicity of “interaction tests” (m = 3), and Benjamini–Hochberg FDR correction was applied with α = 0.05. The raw *p* values were derived from the *p* values of the IADL × years of education interaction term in Model 14. The BH-corrected *p* values were calculated as *p* × (m/i) with monotonicity enforcement. “Yes” for significance after correction indicates that the result remained significant after BH correction, whereas “No” indicates that it was no longer significant after correction. * indicates a raw *p* value that was significant before correction. After correction, only the interaction term for the anti-saccade corrected error rate remained significant, whereas the interaction terms for the uncorrected error rate and correct rate were no longer significant. The above results pertain to the second half-pathway interaction term and cannot replace the moderated mediation index test; in this study, the moderated mediation indices for all outcomes were not statistically supported. The above moderation results are all interpreted in terms of cross-sectional conditional associations.

## Data Availability

The original contributions presented in this study are included in the article/Supplementary Materials. Further inquiries can be directed to the corresponding author.

## Supplementary Materials

The following supporting information can be downloaded at: https://www.mdpi.com/article/10.3390/brainsci16101137/s1, Table S1. Regression analysis of the mediating effect of IADL between MoCA and anti-saccade corrected error rate. Table S2. Test of the oderating effect of years of education in the moderated mediation model (PROCESS Model 7 & Model 14). Table S3. Conditional Effects of IADL on the Anti-Saccade Corrected Error Rate at Different Levels of Years of Education. Table S4. Regression analysis of the mediating effect of IADL between MoCA and anti-saccade correct rate. Table S5. Test of the moderation effect of years of education in the moderated mediation model (PROCESS Model 7 & Model 14). Table S6. Regression Analysis of the Mediating Effect of IADL Between MoCA and the Anti-Saccade Uncorrected Error Rate (MoCA Sensitivity Analysis). Table S7. Test of the Moderating Effect of Years of Education in Moderated Mediation Models (MoCA Sensitivity Analysis). Table S8. Conditional Effects of IADL on the Anti-Saccade Uncorrected Error Rate at Different Levels of Years of Education (MoCA Sensitivity Analysis). Table S9. Regression analysis of the mediating effect of IADL between MoCA and the uncorrected error rate of the antisaccade task (HAMA/HAMD sensitivity analysis). Table S10. Test of the moderating effect of years of education in the moderated mediation models (HAMA/HAMD sensitivity analysis). Table S11. Conditional Effects of IADL on the Anti-Saccade Uncorrected Error Rate Across Different Levels of Years of Education (HAMA/HAMD sensitivity analysis). Figure S1. Histogram of standardized regression residuals for the main mediation model of anti-saccade uncorrected error rate. Figure S2. Normal P-P plot of standardized regression residuals for the main mediation model of anti-saccade uncorrected error rate. Figure S3. Scatter plot of standardized regression residuals for the main mediation model of anti-saccade uncorrected error rate.

## Abbreviations

The following abbreviations are used in this manuscript:

CSVD	Cerebral small vessel disease
WMH	White matter hyperintensities
IADL	Instrumental activities of daily living
dACC	dorsal anterior cingulate cortex
aMCI	amnestic mild cognitive impairment
MoCA	The Montreal Cognitive Assessment
HAMA	Hamilton Anxiety Scale
HAMD	Hamilton Depression Scale
