# Supplementary material for: Instrumental Activities of Daily Living as a Mediator Between Cognition and Anti-Saccade Uncorrected Error Rate in CSVD: An Exploratory Analysis with Education Moderation Testing
Source: Brain Sci. 2026 Oct 21;16(10):1137. doi: 10.3390/brainsci16101137 (PMC13702375; doi:10.3390/brainsci16101137)
Supplement: Supplementary file 1 [file brainsci-16-01137-s001.zip › brainsci-4590964-supplementary.pdf]

S1. Mediation Models Related to Anti-Saccade Corrected Error Rate

S1.1. Mediating Effect of IADL Between MoCA and Anti-Saccade Corrected Error Rate

In this mediation analysis, when the dependent variable was the anti-saccade corrected error rate, its denominator was the number of trials with an initial direction error, and 163 patients were available for computation; 8 patients without initial direction error trials were treated as missing values. PROCESS v4.2 Model 4 was used to test the mediating effect of IADL between MoCA and the anti-saccade corrected error rate (Table S1), with MoCA as the independent variable, IADL as the mediator, and the anti-saccade corrected error rate as the dependent variable, and with age, years of education, and vascular risk factors as covariates.

The results showed that the total effect of MoCA on the anti-saccade corrected error rate was not significant (p = 0.063, 95% CI [-0.073, 2.800]); the predictive effect of MoCA on IADL was significant (p = 0.023, 95% CI [0.007, 0.090]); after controlling for MoCA, the predictive effect of IADL on the anti-saccade corrected error rate was significant (p < 0.001, 95% CI [4.873, 15.369]). After controlling for IADL, the direct effect of MoCA on the anti-saccade corrected error rate was not significant (p = 0.219, 95% CI [-0.526, 2.277]). The Bootstrap results with 5000 resamples showed that the indirect effect of IADL between MoCA and the anti-saccade corrected error rate was not significant (95% BootCI [-0.034, 1.331]).

The results suggest that this pattern is consistent with the indirect association hypothesis that "lower MoCA may be associated with a reduced anti-saccade corrected error rate through IADL impairment," but this indirect effect did not reach statistical significance, and causal mediation cannot be inferred from it. It should be noted that this result describes only the indirect effect estimate for the anti-saccade corrected error rate itself; its confidence interval is close to but does not include zero, and this study did not conduct a formal direct comparison of the indirect effects among the three anti-saccade outcomes. Therefore, this result cannot be directly compared with the significant result for the uncorrected error rate in the main text, nor can it be used to infer outcome-specific mediation or different neural mechanisms.

Table S1. Regression analysis of the mediating effect of IADL between MoCA and anti-saccade corrected error rate.

Effect type	Path	Effect	SE	t	*p*	95% CI
Total effect	MoCA → anti-saccade corrected error rate	1.363	0.727	1.874	0.063	[-0.073, 2.800]
Path a	MoCA → IADL	0.048	0.021	2.293	0.023*	[0.007, 0.090]
Path b	IADL → anti-saccade corrected error rate	10.121	2.657	3.810	<0.001**	[4.873, 15.369]
Direct effect (c')	MoCA → anti-saccade corrected error rate (after controlling for IADL)	0.875	0.710	1.234	0.219	[-0.526, 2.277]
Indirect effect (a × b)	MoCA → IADL → anti-saccade corrected error rate	0.488	0.354#	—	—	[-0.034, 1.331]#

Note: *p < 0.05, **p < 0.01. The indirect effect used Bootstrap with 5000 resamples, BootSE = 0.354, 95% BootCI = [-0.034, 1.331]. The total effect, direct effect, path a, and path b were all derived from the Model 4 output. The anti-saccade corrected error rate was based on data from 163 patients (8 patients without initial direction error trials were treated as missing values). This table presents supplementary exploratory results, and no direct comparison of indirect effects across outcomes was performed.

S1.2. Test of the Moderating Effect of Years of Education in the Mediation Model Involving the Anti-Saccade Corrected Error Rate

PROCESS v4.2 Model 7 and Model 14 were used to test the moderating effects of years of education on the first half-path (MoCA → IADL) and the second half-path (IADL → anti-saccade corrected error rate) of the mediation model, respectively (Table S2), with years of education as the moderator and age and vascular risk factors as covariates.

The results showed that in Model 7, the MoCA × years of education interaction term did not significantly predict IADL (p = 0.771, 95% CI [-0.010, 0.013]); in Model 14, the IADL × years of education interaction term significantly predicted the anti-saccade corrected error rate (p = 0.016, 95% CI [-3.281, -0.346]). The Bootstrap results with 5000 resamples showed that the index of moderated mediation was 0.017 for Model 7 (95% BootCI [-0.182, 0.208]) and -0.073 for Model 14 (95% BootCI [-0.186, 0.016]).

Two types of results need to be clearly distinguished: the significant IADL × years of education interaction term in Model 14 indicates that years of education may moderate the conditional association of the second half-path "IADL → anti-saccade corrected error rate"; whereas the index of moderated mediation tests a different hypothesis, namely "whether the indirect effect varies with years of education." In this study, the confidence intervals of the index of moderated mediation in both models included 0, suggesting that the moderating effect of years of education on the overall mediation effect was not statistically supported.

Therefore, a significant path interaction term cannot be equated with the establishment of a moderated mediation effect. It should be noted that this result describes only the second half-path interaction term and the index of moderated mediation for the anti-saccade corrected error rate itself; this study did not conduct a formal direct comparison of the indirect effects among the three anti-saccade outcomes. Therefore, this result cannot be directly compared with the result for the anti-saccade uncorrected error rate in the main text, nor can it be used to infer outcome-specific moderation or different neural mechanisms.

Table S2. Test of the oderating effect of years of education in the moderated mediation model (PROCESS Model 7 & Model 14).

Model and path	Dependent variable	Predictor	coeff	SE	t	p	95% CI	R^2-change
Model 7	IADL	MoCA × years of education	0.002	0.006	0.292	0.771	[-0.010, 0.013]	0.001
Model 14	Anti-saccade corrected error rate	IADL × years of education	−1.814	0.743	−2.441	0.016*	[−3.281, −0.346]	0.032
Index of moderated mediation (Index)	—	Years of education	Model 7：0.017	0.094[a]	—	—	[−0.182, 0.208][b]	—
Index of moderated mediation (Index)	—	Years of education	Model 14：-0.073	0.052[a]	—	—	[−0.186, 0.016][b]	—

Note: *p < 0.05. In Model 7, MoCA and years of education were mean-centered; in Model 14, IADL and years of education were mean-centered. The number of Bootstrap resamples was 5000. [a] denotes the BootSE; [b] denotes the BootCI; if the confidence interval includes 0, the effect is considered non-significant. The anti-saccade corrected error rate was based on data from 163 patients (8 patients without initial direction error trials were treated as missing values). The significant IADL × years of education interaction term in Model 14 does not indicate that a moderated mediation effect is statistically supported; in this study, the 95% BootCI of the index of moderated mediation in Model 14 included 0. This table did not apply FDR correction to the interaction terms of the three outcomes; the correction results are presented

in Table 8 of the main text. The interaction term for the anti-saccade corrected error rate remained significant after FDR correction, but this result still cannot replace the test of the index of moderated mediation, nor can it be used to infer outcome-specific moderation or different neural mechanisms.

S1.3. Conditional Effects of IADL on Anti-Saccade Corrected Error Rate at Different Levels of Years of Education

To further examine the moderating effect of years of education, simple slope analysis was used to test the conditional effects of IADL on the anti-saccade corrected error rate at different levels of years of education (Table S3).

The results showed that as years of education increased, the absolute value of the positive effect of IADL on the anti-saccade corrected error rate gradually decreased: the effect was 17.485 in the low education group (M - 1 SD, 7.97 years; $p < 0.001$, 95% BootCI [9.597, 25.373]); 11.705 in the medium education group (mean, 11.16 years; $p < 0.001$, 95% BootCI [6.382, 17.028]); and 5.925 in the high education group (M + 1 SD, 14.35 years; $p = 0.060$, 95% BootCI [-0.257, 12.108]). The Johnson–Neyman method showed that when years of education reached 14.25 years, the 95% confidence interval of the conditional effect of IADL on the anti-saccade corrected error rate included 0 for the first time ($p > 0.05$).

It should be noted that the Johnson–Neyman boundary is only used to identify the region in which the confidence interval of the conditional association includes zero; it does not mean that the effect disappears completely or that there is an absolute protective education threshold. In this study sample, the number of participants with years of education exceeding this cutoff was relatively limited, so this value should not be interpreted as a clinical intervention threshold. This analysis addresses only the conditional effect of the second half-path and cannot serve as evidence that the moderated mediation effect is significant; the 95% BootCI of the index of moderated mediation included 0.

Table S3. Conditional Effects of IADL on the Anti-Saccade Corrected Error Rate at Different Levels of Years of Education

Years of education group	Evaluation point (M ± 1 SD)	Effect	SE	t	*p*	95%BootCI
Low education group	M − 1 SD (7.97 years)	17.485	3.993	4.379	<0.001**	[9.597, 25.373]
Medium education group	Mean (11.16 years)	11.705	2.695	4.344	<0.001**	[6.382, 17.028]
High education group	M + 1 SD (14.35 years)	5.925	3.130	1.893	0.060	[-0.257, 12.108]

Note: $^{**}p < 0.01$. The number of Bootstrap resamples was 5000. The effect value represents the absolute percentage-point change in the anti-saccade corrected error rate for each 1-point increase in IADL; a larger positive value indicates a weaker harmful effect of IADL impairment on eye movement inhibition. Years of education were mean-centered in the model, and the evaluation points in the table have been converted back to actual years of education. The Johnson–Neyman method showed that when years of education reached 14.25 years, the 95% confidence interval of the conditional effect of IADL on the anti-saccade corrected error rate included 0 for the first time. It should be noted that this only indicates that the conditional association is no longer statistically significant at this education level; it does not prove that the association disappears completely or that a clinical protective threshold exists. The anti-saccade corrected error rate was based on data from 163 patients.

S2. Mediation Models Related to Anti-Saccade Correct Rate

S2.1. Mediating Effect of IADL Between MoCA and Anti-Saccade Correct Rate

In this mediation analysis, when the dependent variable was the anti-saccade correct rate, its denominator was the number of valid anti-saccade trials, and 171 patients were available for computation. PROCESS v4.2 Model 4 was used to test the mediating effect of IADL between MoCA and the anti-saccade correct rate, with MoCA as the independent variable, IADL as the mediator, and the anti-saccade correct rate as the dependent variable, and with age, years of education, and vascular risk factors as covariates (Table S4).

The results showed that the total effect of MoCA on the anti-saccade correct rate was significant (p = 0.001, 95% CI [0.882, 3.505]); the predictive effect of MoCA on IADL was significant (p = 0.016, 95% CI [0.009, 0.089]); after controlling for MoCA, the predictive effect of IADL on the anti-saccade correct rate was not significant (p = 0.056, 95% CI [-0.128, 9.991]). After controlling for IADL, the direct effect of MoCA on the anti-saccade correct rate remained significant (p = 0.004, 95% CI [0.627, 3.276]). The Bootstrap results with 5000 resamples showed that the indirect effect of IADL between MoCA and the anti-saccade correct rate was not significant (95% BootCI [-0.031, 0.714]).

The results suggest that this pattern is consistent in direction with the indirect association hypothesis that "lower MoCA may be associated with a reduced anti-saccade correct rate through IADL impairment," but this indirect effect did not reach statistical significance, and causal mediation cannot be inferred from it. It should be noted that this result describes only the indirect effect estimate for the anti-saccade correct rate itself; its confidence interval includes zero, and this study did not conduct a formal direct comparison of the indirect effects among the three anti-saccade outcomes. Therefore, this result cannot be directly compared with the significant result for the uncorrected error rate in the main text, nor can it be used to infer outcome-specific mediation or different neural mechanisms.

Table S4. Regression analysis of the mediating effect of IADL between MoCA and anti-saccade correct rate.

Effect type	Path	Effect	SE	t	p	95% CI
Total effect	MoCA → anti-saccade correct rate	2.193	0.664	3.302	0.001**	[0.882, 3.505]
Path a	MoCA → IADL	0.049	0.020	2.440	0.016*	[0.009, 0.089]
Path b	IADL → anti-saccade correct rate	4.932	2.562	1.925	0.056	[-0.128, 9.991]
Direct effect (c')	MoCA → anti-saccade correct rate (after controlling for IADL)	1.952	0.671	2.910	0.004**	[0.627, 3.276]
Indirect effect (a × b)	MoCA → IADL → anti-saccade correct rate	0.242	0.194#	—	—	[-0.031, 0.714]#

Note: *p < 0.05, **p < 0.01. #The indirect effect used Bootstrap with 5000 resamples, BootSE = 0.194, 95% BootCI = [-0.031, 0.714]. The total effect, direct effect, path a, and path b were all derived from the Model 4 output. The anti-saccade correct rate was based on data from 171 patients. This table presents supplementary exploratory results, and no direct comparison of indirect effects across outcomes was performed.

S2.2. Test of the Moderation Effect of Years of Education in the Mediation Model Related to Anti-Saccade Correct Rate

PROCESS v4.2 Model 7 and Model 14 were used to test the moderating effects of years of education on the first half-path (MoCA → IADL) and the second half-path (IADL → anti-

saccade correct rate) of the mediation model, respectively (Table S5), with years of education as the moderator and age and vascular risk factors as covariates.

The results showed that in Model 7, the MoCA × years of education interaction term did not significantly predict IADL (p = 0.698, 95% CI [-0.009, 0.013]); in Model 14, the IADL × years of education interaction term did not significantly predict the anti-saccade correct rate (p = 0.384, 95% CI [-0.806, 2.082]). The Bootstrap results with 5000 resamples showed that the index of moderated mediation was 0.010 for Model 7 (95% BootCI [-0.097, 0.098]) and 0.027 for Model 14 (95% BootCI [-0.052, 0.119]).

The results suggest that the moderating effects of years of education on the first half-path "MoCA → IADL" and the second half-path "IADL → anti-saccade correct rate" were both non-significant; the confidence intervals of the index of moderated mediation in both models included 0, suggesting that the moderating effect of years of education on the overall mediation effect was not statistically supported. Therefore, in this model, neither the path interaction terms nor the index of moderated mediation can be interpreted as evidence that the moderating effect of education is established, and conditional effects at different levels of years of education are no longer reported. It should be noted that this result describes only the first half-path, second half-path interaction terms, and the index of moderated mediation for the anti-saccade correct rate itself; this study did not conduct a formal direct comparison of the indirect effects or moderating effects among the three anti-saccade outcomes. Therefore, this result cannot be directly compared with the results for the anti-saccade uncorrected error rate in the main text or the anti-saccade corrected error rate in the supplementary materials, nor can it be used to infer outcome-specific moderation or different neural mechanisms. This result is consistent with the conclusion in Table 8 of the main text that the interaction term for the anti-saccade correct rate remained non-significant after FDR correction.

Table S5. Test of the moderation effect of years of education in the moderated mediation model (PROCESS Model 7 & Model 14).

Model and path	Dependent variable	Predictor	coeff	SE	t	p	95% CI	R^2-change
Model 7	IADL	MoCA × years of education	0.002	0.006	0.389	0.698	[-0.009, 0.013]	0.001
Model 14	Anti-saccade correct rate	IADL × years of education	0.638	0.731	0.872	0.384	[-0.806, 2.082]	0.004
Index of moderated mediation (Index)	—	Years of education	Model 7：0.010	0.046[a]	—	—	[-0.097, 0.098][b]	—
Index of moderated mediation (Index)	—	Years of education	Model 14：0.027	0.041[a]	—	—	[-0.052, 0.119][b]	—

Note: All models controlled for age and vascular risk factors; in Model 7, MoCA and years of education were mean-centered; in Model 14, IADL and years of education were mean-centered. The number of Bootstrap resamples was 5000. [a] denotes the BootSE; [b] denotes the BootCI; if the confidence interval includes 0, the effect is considered non-significant. The anti-saccade correct rate was based on data from 171 patients. In this table, the interaction terms and the index of moderated mediation in both Model 7 and Model 14 were non-significant, suggesting that the moderating effects of years of education on the first half-path, the second half-path, and the overall mediation effect were not statistically supported. This table did not apply FDR correction to the interaction terms of the three outcomes; the correction results are presented in Table 8 of the main text. The interaction term for the anti-saccade correct rate remained non-significant after FDR correction. This table presents supplementary exploratory results,

and no direct comparison of moderating effects or indirect effects across outcomes was performed; therefore, it cannot be used to infer outcome-specific moderation or different neural mechanisms.

S3. MoCA Sensitivity Analysis

S3.1. Mediating Effect of IADL Between MoCA and Anti-Saccade Uncorrected Error Rate (MoCA Sensitivity Analysis)

In this sensitivity analysis, when the dependent variable was the anti-saccade uncorrected error rate, its denominator was the number of valid anti-saccade trials, and 171 patients were available for computation. To further test the robustness of the main analysis results, PROCESS v4.2 Model 4 was used to test the mediating effect of IADL between the MoCA raw score and the anti-saccade uncorrected error rate (Table S6), with the MoCA raw score as the independent variable, IADL as the mediator, and the anti-saccade uncorrected error rate as the dependent variable, and with age, years of education, and vascular risk factors as covariates. The settings for other variables and covariates were consistent with the main analysis.

The results showed that the total effect of the MoCA raw score on the anti-saccade uncorrected error rate was significant ($p = 0.003$, 95% CI [-2.396, -0.484]); the predictive effect of the MoCA raw score on IADL was significant ($p = 0.019$, 95% CI [0.008, 0.087]); after controlling for the MoCA raw score, the predictive effect of IADL on the anti-saccade uncorrected error rate was significant ($p < 0.001$, 95% CI [-12.214, -5.268]). After controlling for IADL, the direct effect of the MoCA raw score on the anti-saccade uncorrected error rate remained significant ($p = 0.027$, 95% CI [-1.933, -0.116]). The Bootstrap results with 5000 resamples showed that the indirect effect of IADL between the MoCA raw score and the anti-saccade uncorrected error rate was significant (95% BootCI [-1.119, -0.006]).

The results suggest that this pattern is consistent with the indirect association hypothesis that "a lower MoCA raw score may be associated with a higher anti-saccade uncorrected error rate through IADL impairment." The sensitivity analysis results were basically consistent with the main analysis, but causal mediation cannot be inferred from them.

Table S6. Regression Analysis of the Mediating Effect of IADL Between MoCA and the Anti-Saccade Uncorrected Error Rate (MoCA Sensitivity Analysis).

Effect type	Path	Effect	SE	t	*p*	95% CI
Total effect	MoCA → anti-saccade uncorrected error rate	-1.440	0.484	-2.974	0.003**	[-2.396, -0.484]
Path a	MoCA → IADL	0.048	0.020	2.364	0.019*	[0.008, 0.087]
Path b	IADL → anti-saccade uncorrected error rate	-8.741	1.759	-4.970	<0.001**	[-12.214, -5.268]
Direct effect (c')	MoCA → anti-saccade uncorrected error rate (after controlling for IADL)	-1.025	0.460	-2.227	0.027*	[-1.933, -0.116]
Indirect effect (a × b)	MoCA → IADL → anti-saccade uncorrected error rate	-0.415	0.297#	—	—	[-1.119, -0.006]#

Note: *$p < 0.05$, **$p < 0.01$. #The indirect effect used Bootstrap with 5000 resamples, BootSE = 0.297, 95% BootCI = [-1.119, -0.006]. The total effect, direct effect, path a, and path b were all derived from the Model 4 output. The anti-saccade uncorrected error rate was based on data from 171 patients. This table presents sensitivity analysis results with the MoCA raw score as the independent variable, and the settings for other variables and covariates were consistent with the main analysis.

S3.2. Test of the Moderating Effect of Years of Education in Moderated Mediation Models (MoCA Sensitivity Analysis)

In this sensitivity analysis, when the dependent variable was the anti-saccade uncorrected error rate, its denominator was the number of valid anti-saccade trials, and 171 patients were available for computation. To further test the robustness of the moderating effect in the main analysis, PROCESS v4.2 Model 7 and Model 14 were used to test the moderating effects of years of education on the first half-path (MoCA raw score → IADL) and the second half-path (IADL → anti-saccade uncorrected error rate) of the mediation model, respectively (Table S7), with years of education as the moderator and age and vascular risk factors as covariates. The settings for other variables and covariates were consistent with the main analysis.

The results showed that in Model 7, the MoCA raw score × years of education interaction term did not significantly predict IADL (p = 0.870, 95% CI [-0.010, 0.012]); in Model 14, the IADL × years of education interaction term significantly predicted the anti-saccade uncorrected error rate (p = 0.035, 95% CI [0.077, 2.040]). The Bootstrap results with 5000 resamples showed that the index of moderated mediation was -0.007 for Model 7 (95% BootCI [-0.165, 0.119]) and 0.039 for Model 14 (95% BootCI [-0.019, 0.112]).

Two types of results need to be clearly distinguished: the significant IADL × years of education interaction term in Model 14 indicates that years of education may moderate the conditional association of the second half-path "IADL → anti-saccade uncorrected error rate"; whereas the index of moderated mediation tests a different hypothesis, namely "whether the indirect effect varies with years of education." In this study, the confidence intervals of the index of moderated mediation in both models included 0, suggesting that the moderating effect of years of education on the overall mediation effect was not statistically supported. Therefore, a significant path interaction term cannot be equated with the establishment of a moderated mediation effect. It should be noted that this sensitivity analysis addressed only the anti-saccade uncorrected error rate and was not used to infer differences in indirect effects or moderating effects among the three anti-saccade outcomes.

Table S7. Test of the Moderating Effect of Years of Education in Moderated Mediation Models (MoCA Sensitivity Analysis).

Model and path	Dependent variable	Predictor	coeff	SE	t	p	95% CI	R^2-change
Model 7	IADL	MoCA × years of education	0.001	0.006	0.164	0.870	[-0.010, 0.012]	0.000
Model 14	Anti-saccade uncorrected error rate	IADL × years of education	1.058	0.497	2.130	0.035*	[0.077, 2.040]	0.022
Index of moderated mediation (Index)	—	Years of education	Model 7: -0.007	0.070[a]	—	—	[-0.165, 0.119][b]	—
Index of moderated mediation (Index)	—	Years of education	Model 14: 0.039	0.033[a]	—	—	[-0.019, 0.112][b]	—

Note: *p < 0.05. In Model 7, MoCA and years of education were mean-centered; in Model 14, IADL and years of education were mean-centered. The number of Bootstrap resamples was 5000. [a] denotes the BootSE; [b] denotes the BootCI; if the confidence interval includes 0, the effect is considered non-significant. The anti-saccade uncorrected error rate was based on data from 171 patients. This table presents sensitivity analysis results with the MoCA raw score as the independent variable, and the settings for other variables and covariates were consistent with the main analysis. The significant IADL × years of education interaction term in Model 14 only suggests that the conditional association of the second half-

path may vary with years of education; it does not indicate that a moderated mediation effect is statistically supported. In this study, the 95% BootCI of the index of moderated mediation in Model 14 included 0.

S3.3. Conditional Effects of IADL on the Anti-Saccade Uncorrected Error Rate at Different Levels of Years of Education (MoCA Sensitivity Analysis)

To further examine the moderating effect of years of education, simple slope analysis was used to test the conditional effects of IADL on the anti-saccade uncorrected error rate at different levels of years of education (Table S8).

The results showed that as years of education increased, the absolute value of the negative effect of IADL on the anti-saccade uncorrected error rate gradually decreased: the effect was -12.930 in the low education group (M - 1 SD, 8.03 years; $p < 0.001$, 95% BootCI [-18.115, -7.745]); -9.615 in the medium education group (mean, 11.16 years; $p < 0.001$, 95% BootCI [-13.145, -6.085]); and -6.299 in the high education group (M + 1 SD, 14.29 years; $p = 0.003$, 95% BootCI [-10.414, -2.184]). The Johnson–Neyman method showed that when years of education reached 15.59 years, the 95% confidence interval of the conditional effect of IADL on the anti-saccade uncorrected error rate included 0 for the first time ($p \geq 0.05$).

It should be noted that the Johnson–Neyman boundary is only used to identify the region in which the confidence interval of the conditional association includes zero; it does not mean that the effect disappears completely or that there is an absolute protective education threshold. In this study sample, the number of participants with years of education exceeding this cutoff was relatively limited, so this value should not be interpreted as a clinical intervention threshold. This analysis addresses only the conditional effect of the second half-path and cannot serve as evidence that the moderated mediation effect is significant; the 95% BootCI of the index of moderated mediation included 0.

Table S8. Conditional Effects of IADL on the Anti-Saccade Uncorrected Error Rate at Different Levels of Years of Education (MoCA Sensitivity Analysis)

Years of education group	Evaluation point (M ± 1 SD)	Effect	SE	t	*p*	95%BootCI
Low education group	M − 1 SD (8.03 years)	-12.930	2.626	-4.924	<0.001**	[-18.115, -7.745]
Medium education group	Mean (11.16 years)	-9.615	1.788	-5.378	<0.001**	[-13.145, -6.085]
High education group	M + 1 SD (14.29 years)	-6.299	2.084	-3.023	0.003**	[-10.414, -2.184]

Note: $*p < 0.05$, $**p < 0.01$. The number of Bootstrap resamples was 5000. The effect value represents the absolute percentage-point change in the anti-saccade uncorrected error rate for each 1-point increase in IADL; a larger absolute value of the negative effect indicates a stronger harmful effect of IADL impairment on eye movement inhibition. Years of education were mean-centered in the model, and the evaluation points in the table have been converted back to actual years of education. The anti-saccade uncorrected error rate was based on data from 171 patients. This table presents sensitivity analysis results with the MoCA raw score as the independent variable.

S4. HAMA/HAMD Sensitivity Analysis

S4.1. Mediating effect of IADL between MoCA and the uncorrected error rate of the antisaccade task (HAMA/HAMD sensitivity analysis)

In this sensitivity analysis, when the dependent variable was the uncorrected error rate of the antisaccade task, its denominator was the number of valid antisaccade trials, and the number of patients available for analysis was 171. To further examine the robustness of the main analysis results, Model 4 of PROCESS 4.2 was used to test the mediating effect of IADL between MoCA and the uncorrected error rate of the antisaccade task (Table S9). Age, years of education, vascular risk factors, HAMA, and HAMD were included as covariates, with other variable specifications consistent with the main analysis.

The results showed that the total effect of MoCA on the uncorrected error rate of the antisaccade task was significant (p = 0.005, 95% CI [−2.356, −0.436]); the predictive effect of MoCA on IADL was significant (p = 0.045, 95% CI [0.001, 0.076]); after controlling for MoCA, the predictive effect of IADL on the uncorrected error rate of the antisaccade task was significant (p < 0.001, 95% CI [−12.148, −4.663]). After controlling for IADL, the direct effect of MoCA on the uncorrected error rate of the antisaccade task remained significant (p = 0.023, 95% CI [−1.992, −0.151]). Bootstrap results with 5000 resamples showed that the indirect effect of IADL between MoCA and the uncorrected error rate of the antisaccade task was not significant (95% BootCI [−1.064, 0.080]).

These results suggest that this pattern is inconsistent with the indirect association hypothesis that "lower MoCA may be associated with a higher uncorrected error rate of the antisaccade task through impaired IADL"; however, causal mediation cannot be inferred on this basis.

Table S9 Regression analysis of the mediating effect of IADL between MoCA and the uncorrected error rate of the antisaccade task (HAMA/HAMD sensitivity analysis)

Effect type	Path	Effect	SE	t	p	95% CI
Total effect	MoCA → anti-saccade uncorrected error rate	-1.396	0.486	-2.872	0.005**	[-2.356, -0.436]
Path a	MoCA → IADL	0.039	0.019	2.023	0.045*	[0.001, 0.076]
Path b	IADL → anti-saccade uncorrected error rate	-8.406	1.895	-4.435	<0.001**	[-12.148, -4.663]
Direct effect (c')	MoCA → anti-saccade uncorrected error rate (after controlling for IADL)	-1.071	0.466	-2.299	0.023*	[-1.992, -0.151]
Indirect effect (a × b)	MoCA → IADL → anti-saccade uncorrected error rate	-0.324	0.302#	—	—	[-1.064, 0.080]#

Note: *p < 0.05, **p < 0.01. #The indirect effect was estimated using 5000 bootstrap resamples, BootSE = 0.302, 95% BootCI = [−1.064, 0.080]. The total effect, direct effect, path a, and path b were all derived from the Model 4 output. The uncorrected error rate of the antisaccade task was based on data from 171 patients. This table presents the results of the sensitivity analysis with HAMA and HAMD added as covariates, with other variable specifications consistent with the main analysis.

S4.2. Test of the moderating effect of years of education in the moderated mediation models (HAMA/HAMD sensitivity analysis)

In this sensitivity analysis, when the dependent variable was the uncorrected error rate of the antisaccade task, its denominator was the number of valid antisaccade trials, and the number of patients available for analysis was 171. To further examine the robustness of the

moderating effects in the main analysis, Model 7 and Model 14 of PROCESS 4.2 were used to test the moderating role of years of education on the first stage (MoCA → IADL) and the second stage (IADL → uncorrected error rate of the antisaccade task) of the mediation model, respectively (Table S10). Age, years of education, vascular risk factors, HAMA, and HAMD were included as covariates, with other variable specifications consistent with the main analysis.

The results showed that in Model 7, the MoCA × years of education interaction term did not significantly predict IADL ($p = 0.642$, 95% CI [−0.008, 0.013]); in Model 14, the IADL × years of education interaction term significantly predicted the uncorrected error rate of the antisaccade task ($p = 0.040$, 95% CI [0.048, 2.052]). Bootstrap results with 5000 resamples showed that the index of moderated mediation was −0.019 (95% BootCI [−0.187, 0.110]) for Model 7 and 0.031 (95% BootCI [−0.034, 0.107]) for Model 14.

It is necessary to clearly distinguish between these two types of results: the significant IADL × years of education interaction term in Model 14 indicates that years of education may moderate the conditional association of the second stage, "IADL → uncorrected error rate of the antisaccade task"; whereas the index of moderated mediation tests a different hypothesis, namely whether the indirect effect varies with years of education. In this study, the confidence intervals of the indices of moderated mediation in both models included 0, indicating that the moderating effect of years of education on the overall mediation effect was not statistically supported. Therefore, a significant path interaction term cannot be equated with the establishment of a moderated mediation effect. It should be noted that this sensitivity analysis was conducted only for the uncorrected error rate of the antisaccade task and should not be used to infer differences in indirect effects or moderating effects among the three antisaccade outcomes.

Table S10 Test of the moderating effect of years of education in the moderated mediation models (HAMA/HAMD sensitivity analysis)

**Model and path**	**Dependent variable**	**Predictor**	**coeff**	**SE**	**t**	***p***	**95% CI**	**R^2-change**
Model 7	IADL	MoCA × years of education	0.003	0.005	0.465	0.642	[-0.008, 0.013]	0.001
Model 14	Anti-saccade uncorrected error rate	IADL × years of education	1.050	0.507	2.069	0.040*	[0.048, 2.052]	0.020
Index of moderated mediation (Index)	—	Years of education	Model 7: -0.019	0.072[a]	—	—	[-0.187, 0.110][b]	—
Index of moderated mediation (Index)	—	Years of education	Model 14: 0.031	0.035[a]	—	—	[-0.034, 0.107][b]	—

Note: *$p < 0.05$. In Model 7, MoCA and years of education were mean-centered; in Model 14, IADL and years of education were mean-centered. The number of bootstrap resamples was 5000. [a] denotes BootSE; [b] denotes [BootLLCI, BootULCI]; if the confidence interval includes 0, the effect is considered non-significant. The uncorrected error rate of the antisaccade task was based on data from 171 patients. This table presents the results with age, years of education, vascular risk factors, HAMA, and HAMD as covariates, with other variable specifications consistent with the main analysis. The significant IADL × years of education interaction term in Model 14 only suggests that the conditional association of the second stage may vary with years of education; it does not indicate that a moderated mediation effect is statistically supported. In this study, the 95% BootCI of the index of moderated mediation in Model 14 included 0.

S4.3. Conditional effect of IADL on the uncorrected error rate of the antisaccade task at different levels of years of education (HAMA/HAMD sensitivity analysis)

To further elucidate the moderating role of years of education, simple slope analysis was used to examine the conditional effect of IADL on the uncorrected error rate of the antisaccade task at different levels of years of education (Table S11).

The results showed that, as years of education increased, the absolute value of the negative effect of IADL on the uncorrected error rate of the antisaccade task gradually decreased: the effect was −12.768 in the low-education group (M − 1 SD, 8.03 years) ($p < 0.001$, 95% BootCI [−18.342, −7.194]); −9.479 in the medium-education group (mean, 11.16 years) ($p < 0.001$, 95% BootCI [−13.324, −5.635]); and −6.190 in the high-education group (M + 1 SD, 14.29 years) ($p = 0.003$, 95% BootCI [−10.457, −1.924]). The Johnson-Neyman method showed that when years of education reached 15.47 years, the 95% confidence interval of the conditional effect of IADL on the uncorrected error rate of the antisaccade task included 0 for the first time (i.e., $p \geq 0.05$).

It should be noted that the Johnson-Neyman boundary is used only to identify the region in which the confidence interval of the conditional association includes zero; it does not mean that the effect disappears entirely or that there is an absolute protective educational threshold. In the present sample, the number of participants with years of education exceeding this threshold was relatively limited; therefore, this value should not be interpreted as a clinical cutoff for intervention. This analysis addresses only the conditional effect of the second stage and cannot serve as evidence that the moderated mediation effect is significant; the 95% BootCI of the index of moderated mediation included 0.

Table S11. Conditional Effects of IADL on the Anti-Saccade Uncorrected Error Rate Across Different Levels of Years of Education (HAMA/HAMD sensitivity analysis)

Years of education group	Evaluation point (M ± 1 SD)	Effect	SE	t	*p*	95%BootCI
Low education group	M − 1 SD (8.03 years)	-12.768	2.822	-4.524	<0.001**	[-18.342, -7.194]
Medium education group	Mean (11.16 years)	-9.479	1.947	-4.870	<0.001**	[-13.324, -5.635]
High education group	M + 1 SD (14.29 years)	-6.190	2.160	-2.866	0.005**	[-10.457, -1.924]

Note: $^{**}p < 0.01$. The number of bootstrap resamples was 5000. The effect value represents the absolute percentage-point change in the uncorrected error rate of the antisaccade task for each 1-point increase in IADL; a larger absolute value of the negative effect indicates a stronger detrimental effect of impaired IADL on oculomotor inhibition. Years of education was mean-centered in the model, and the evaluation points in the table have been converted back to actual years of education. The uncorrected error rate of the antisaccade task was based on data from 171 patients. This table presents the results with age, years of education, vascular risk factors, HAMA, and HAMD as covariates, with other variable specifications consistent with the main analysis.

Figure S1. Histogram of standardized regression residuals for the main mediation model of anti-saccade uncorrected error rate.

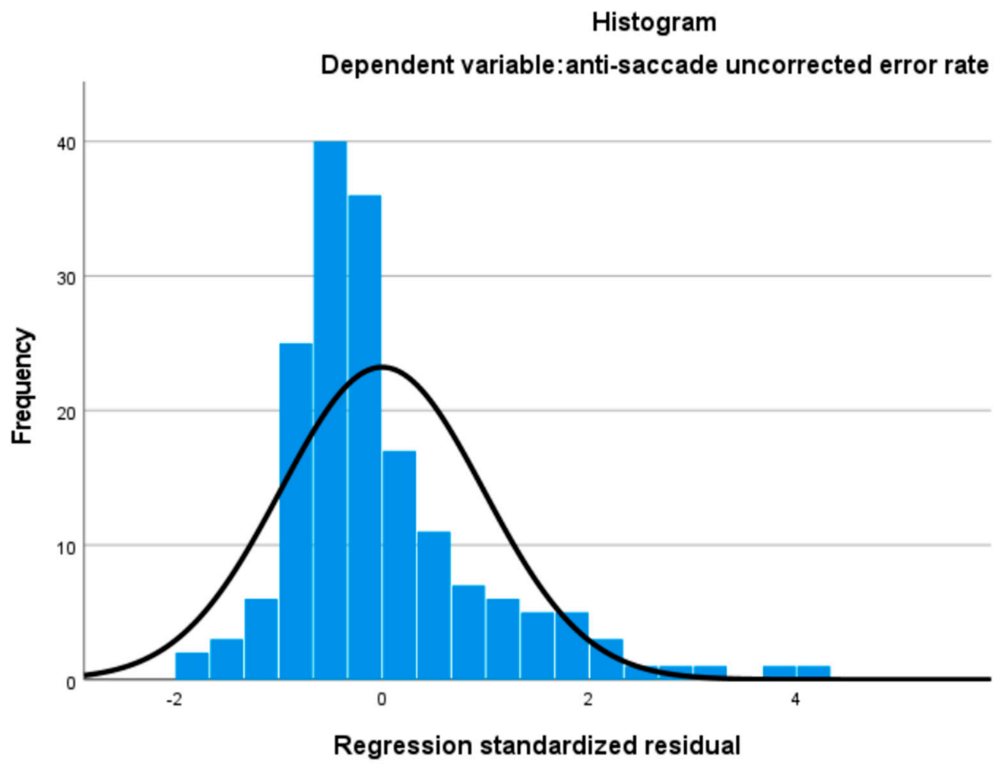

Note: This figure is based on the Model 4 main mediation model in Section 3.3 of the main text, with anti-saccade uncorrected error rate as the dependent variable, MoCA as the independent variable, IADL as the mediator, and age, years of education, and vascular risk factors as covariates. The figure shows that the residual distribution presents a very obvious right skew (positive skew).

Figure S2. Normal P-P plot of standardized regression residuals for the main mediation model of anti-saccade uncorrected error rate.

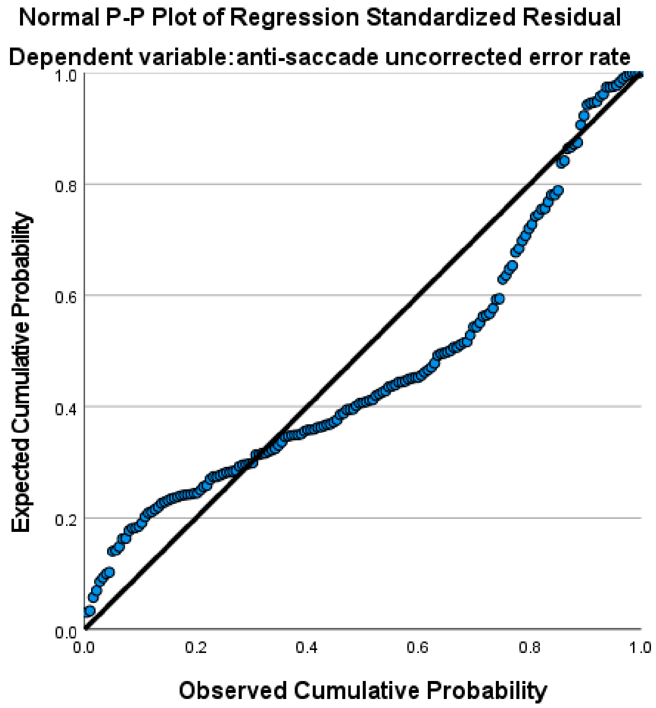

Note: This figure is based on the main mediation model of anti-saccade uncorrected error rate in Section 3.3 of the main text. In the figure, the dotted line deviates from the diagonal in a stepped manner in the middle segment, reflecting residual non-normality caused by the high proportion of zero values.

Figure S3. Scatter plot of standardized regression residuals for the main mediation model of anti-saccade uncorrected error rate.

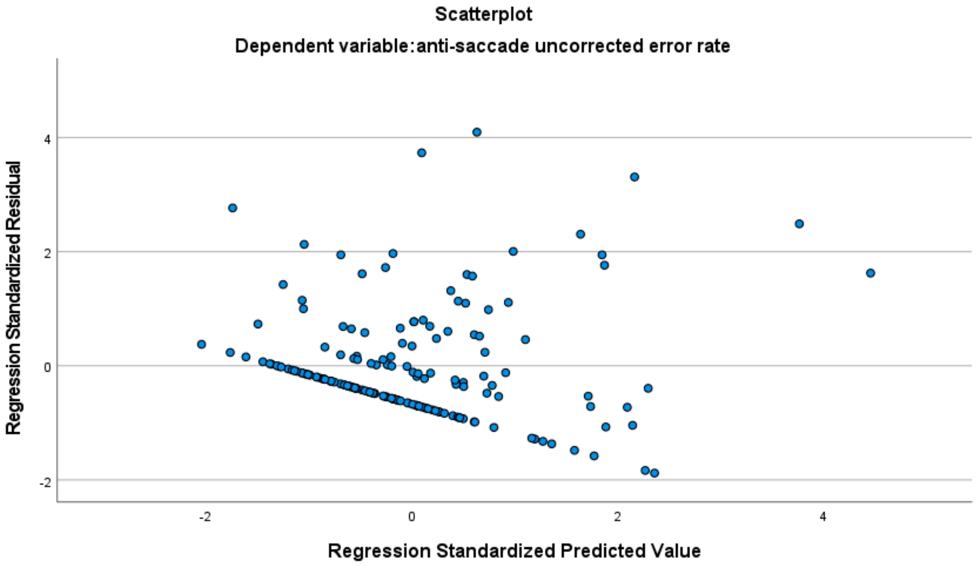

Note: This figure is based on the main mediation model of anti-saccade uncorrected error rate in Section 3.3 of the main text. The figure shows an obvious "fan" or "funnel" shape, suggesting the presence of heteroscedasticity.
